# Blinking characteristics of organic fluorophores for blink-based multiplexing

**DOI:** 10.1038/s42004-024-01106-5

**Published:** 2024-01-27

**Authors:** Amelia G. Seabury, Alisha J. Khodabocus, Isabelle M. Kogan, Grayson R. Hoy, Grace A. DeSalvo, Kristin L. Wustholz

**Affiliations:** Chemistry Department, William & Mary, Williamsburg, VA USA

**Keywords:** Fluorescence spectroscopy, Imaging studies

## Abstract

Single-molecule fluorescence experiments have transformed our understanding of complex materials and biological systems. Whether single molecules are used to report on their nano-environment or provide for localization, understanding their blinking dynamics (i.e., stochastic fluctuations in emission intensity under continuous illumination) is paramount. We recently demonstrated another use for blinking dynamics called blink-based multiplexing (BBM), where individual emitters are classified using a single excitation laser based on blinking dynamics, rather than color. This study elucidates the structure-activity relationships governing BBM performance in a series of model rhodamine, BODIPY, and anthraquinone fluorophores that undergo different photo-physical and-chemical processes during blinking. Change point detection and multinomial logistic regression analyses show that BBM can leverage spectral fluctuations, electron and proton transfer kinetics, as well as photostability for molecular classification—even within the context of a shared blinking mechanism. In doing so, we demonstrate two- and three-color BBM with ≥ 93% accuracy using spectrally-overlapped fluorophores.

## Abstract (web summary)

Single-molecule fluorescent probes can be used for nanoreporting, localization, and now multiplexing, but understanding their stochastic fluctuations in emission intensity is crucial for accurate signal interpretation. Here, the authors elucidate the blinking dynamics of rhodamine, BODIPY, and antraquinone dyes, demonstrate that multiplexing performance improves with photophysical differences, and suggest guidelines for the selection and design of organic fluorophores for single-molecule multiplexing.

## Introduction

By removing the effects of ensemble averaging, single-molecule spectroscopy (SMS) measurements can reveal the distributions of molecular behavior in complex environments such as cells, polymers, and nanomaterials^1–4^. In doing so, single molecules act as “nanoreporters” to probe environmental heterogeneity, detect rare intermediates and hidden kinetic pathways, and track molecules in motion. SMS studies have also led to the invention of super-resolution imaging using single-molecule localization microscopy (SMLM), where individual fluorophores are switched “on” randomly in space, localized, and turned “off” in order to generate a sub-diffraction image of an object^5–8^. In all cases, whether single molecules are used for nanoreporting or localization, understanding their blinking dynamics (i.e., the stochastic fluctuations in emission intensity that occur during continuous illumination due to the population of optically bright and dark vibronic states) is paramount. For example, the durations of emissive (on) and non-emissive (off) events in a blinking trace can be used to report on the dark-state production and decay of a molecule within its local environment^9–11^. On the other hand, blinking and spectral diffusion (i.e., time-dependent changes in a molecule’s absorption spectrum due to environmental and/or photochemical effects) are detrimental to tracking and most SMLM implementations^5,12^.

We recently showed that blinking can be harnessed for a new purpose in single-molecule experiments—identification—in a technique called blink-based multiplexing (BBM)^13,14^. Püntener and Rivera-Fuentes also recently demonstrated a “blinkognition” concept, where the spontaneous blinking of a silicon rhodamine is used to identify peptide structure using deep learning^15^. Multiplexing in single-molecule experiments is typically connected to differences in probe absorption and/or emission spectra^16–24^, fluorescence activation wavelength^20,23^, binding kinetics^25–27^, or through sequential imaging of the same fluorophore^28–30^. The main advantage of BBM is that multiplexed detection of individual emitters is achieved simultaneously, using a single excitation laser, and without probing spectral color. Thus, BBM holds the promise to expand the palette of available probes for SMS and SMLM experiments as well as enhance the versatility of these tools for biological and materials investigations. In the first demonstration of BBM, we showed that the blinking dynamics of two spectrally-indistinct emitters [i.e., rhodamine 6 G (R6G) and core–shell CdSe/ZnS quantum dots (QD)] are sufficiently different to provide for >93% multiplexing accuracy using an empirically-derived metric^13^. More recently, rapid and accurate BBM of R6G and QD in a polymer environment and under a variety of experimental conditions (i.e., bin time, excitation power) was achieved using machine learning^14^. Although these initial method development studies established the feasibility of BBM, several challenges remain to be addressed. For example, it is essential to determine if BBM can differentiate among molecular fluorophores, which are preferable for single-molecule studies due to their small size and biocompatibility^5,31,32^. Furthermore, the relationships among probe structure, fluorescence and spectral properties, blinking mechanism(s), and BBM performance are unknown.

**Fig. 1:**
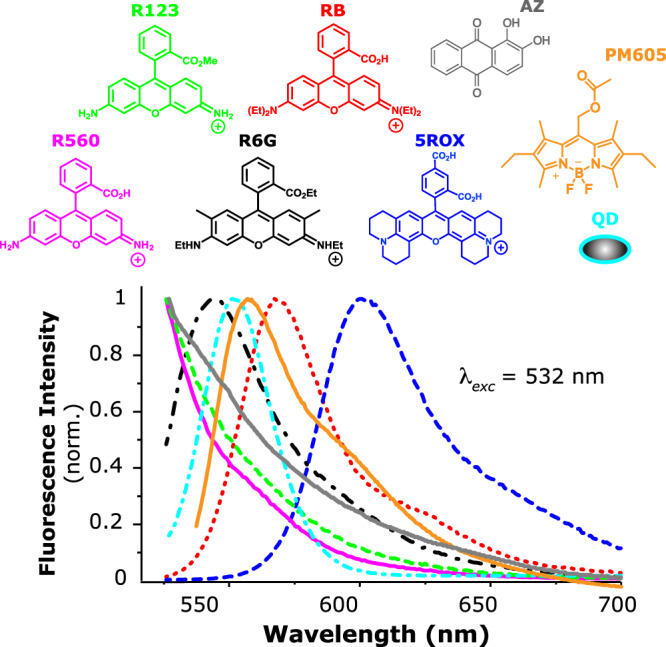
Structures and corresponding ensemble-averaged fluorescence spectra of all probes examined in this study. (pink solid) R560, (green dashed) R123, (black dash-dotted) R6G, (red dotted) RB, (blue dashed) 5ROX, (gray solid) AZ, (cyan dash-dotted) QD, and (orange solid) PM605. Emission spectra obtained using 532-nm excitation are appreciably overlapped, with maxima at ~535 nm for R560, R123, and AZ, 551 nm for R6G, 558 nm for QD, 565 nm for PM605, 574 nm for RB, and 605 nm for 5ROX. Spectra obtained in aqueous or ethanolic solution.

This study establishes how molecular structure and photophysics govern BBM in a series of seven small-molecule probes from three different molecular classes. First, to test the fundamental limits of BBM from a molecular perspective, we examine the blinking dynamics and multiplexing performance among a set of spectrally-overlapped rhodamine dyes: 5-carboxy-X-rhodamine (5ROX), rhodamine 123 (R123), rhodamine 560 (R560, also known as rhodamine 110), rhodamine 6 G (R6G), and rhodamine B (RB). These common SMS probes have well-characterized fluorescence properties^33–37^ and are known to share a common blinking mechanism. Indeed, previous SMS studies of xanthene dyes immobilized on glass observed broad distributions of on and off events ranging from tens of milliseconds to hundreds of seconds in duration, which were attributed to dispersive electron transfer (ET) to/from trap states^38–40^. That is, the on-off switching behavior of 5ROX, R123, R560, R6G, and RB due to ET is highly dispersive, which should hinder BBM. Yet, these dyes also possess structural differences (Fig. 1) that influence their conformational flexibility, polarity sensitivity, as well as propensity to undergo photoinduced N-dealkylation also known as photobluing^32,35,41,42^. Each of these effects can manifest during blinking (e.g., as intensity fluctuations due to spectral shifts) and may either benefit or impede BBM. Therefore, 5ROX, R123, R560, R6G, and RB represent an excellent model system to elucidate how structure, fluorescence and spectral properties, ET kinetics, and corresponding dispersion govern BBM within the context of a shared blinking mechanism.

To determine how additional structural and photophysical changes manifest in blinking and BBM performance, we investigate probes from different classes – a dye from the di-pyrromethene (i.e., BODIPY) family that has generated much interest from the single-molecule community^31,43–49^, but whose blinking mechanism is unknown, an anthraquinone fluorophore that undergoes excited-state intramolecular proton transfer (ESIPT)^50,51^, and QD emitters that exhibit dispersive charge transfer processes^52,53^. In doing so, this study reveals both fundamental insight about blinking as well as useful advancements to the BBM technique. First, despite broad photophysical distributions and a shared on-off blinking mechanism, BBM via machine learning can accurately differentiate spectrally-overlapped rhodamines with ≥ 90% accuracy, though at the expense of data loss. For these dyes, BBM performance is attributed to differences in their ET kinetics and susceptibility to spectral diffusion. By examining the blinking characteristics of PM605, AZ, and QD, we find that differences in on-off switching mechanism, associated kinetics, as well as photostability can also be leveraged for BBM. If the photo-physical and/or -chemical differences among probes are amplified, then overall BBM performance in terms of accuracy and data retention is substantially improved. Indeed, this study demonstrates two- and three-color BBM with ≥ 93% accuracy and modest data loss using spectrally-overlapped, small-molecule probes – even when substantial dispersion exists. Collectively, by elucidating the fundamental relationships among molecular structure, blinking dynamics, and multiplexing capability, this study reveals strategies for the selection and design of BBM probes for further SMS and SMLM studies of spectrally-overlapped fluorophores.

## Results and discussion

### Blinking characteristics and BBM of rhodamine fluorophores

To elucidate the structure-activity relationships governing BBM in a set of fluorophores that share a common blinking mechanism using a single 532-nm excitation laser, we studied 5ROX, R123, R560, R6G, and RB. Figure 1 shows the bulk fluorescence spectra of these dyes following 532-nm excitation to be appreciably overlapped, with maxima well below the ~100 nm separation needed for conventional spectral-based multiplexing^5,20,24^. For example, R560 and 5ROX are the most spectrally distinct, with emission maxima corresponding to ~535 nm and 605 nm, respectively. The other rhodamines exhibit emission maxima shifted by ≤ 30 nm from each other. Figure S1 in the Supplementary Information shows corresponding absorption spectra of these fluorophores, all of which can be photoexcited at 532 nm to varying degrees [i.e., the extinction coefficient at 532 nm (*ε*_532_) ranges from approximately 15,000 to 115,000 M^-1^cm^-1^]^34–39^. In addition to being selected for their spectral overlap and shared blinking mechanism, RB and its close structural analog, R560, are also included in this study as a negative control. That is, previous studies have shown that RB undergoes stepwise photoinduced N-dealkylation at 532 nm to produce R560^42,54,55^. Therefore, RB and R560 emitters should not be appreciably distinguishable with BBM under these experimental conditions because the RB dataset is likely to be polluted with R560 and associated photoproducts.

**Fig. 2:**
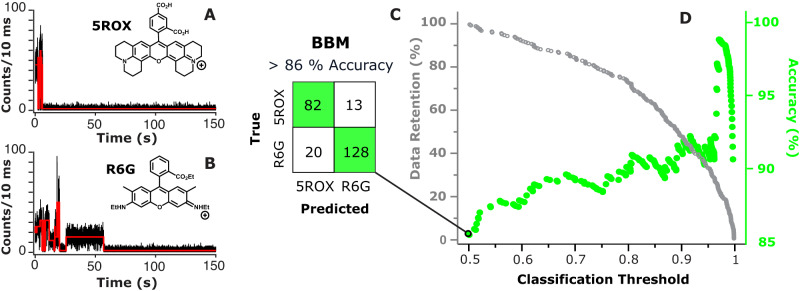
Blinking analysis and MLR-based classification of 5ROX and R6G. Representative blinking dynamics of **A** 5ROX and **B** R6G obtained using 532-nm excitation, 1 $\mu$W power, and 10-ms bin time shown with (red line) CPD analysis. The *N*_*I*_, *I*_*min*_, 〈*I*〉_*t*_, and *N*_*off*,*seg*_ values are 5, 2.6, 2.9, and 4 for the 5ROX molecule and 7, 3.2, 6.9, and 6 for the R6G molecule, respectively, consistent with the associated averages for many molecules. **C** Confusion matrix of true vs. predicted values using BBM for a total of 243 5ROX and R6G molecules yields a minimum classification accuracy of 86.4%. **D** As the classification threshold is raised and more “uncertain” emitters are discarded, (green, circles) overall classification accuracy is increased at the expense of (gray, open circles) data retention. For example, 90% accurate binary classification of 5ROX and R6G is achieved using a classification threshold of 0.66 and corresponding 88% data retention.

The first step in single-molecule blinking studies is to measure the emission-time traces of many molecules and quantify their dynamics using a statistically-robust approach. To this end, blinking measurements of ~100 molecules of 5ROX, R123, R560, R6G, and RB were performed on a confocal microscope under the same experimental conditions – continuous excitation at 532 nm with 1 μW power, 10-ms bin time, and on glass in anoxic environment. Figure 2A, B show representative blinking traces of 5ROX and R6G molecules with corresponding blinking analysis overlaid in red. Consistent with prior studies^10,38,39,56^, the blinking dynamics of these molecules appears to be non-binary and complex, with multiple emissive intensities and event timescales evident within each trace. Since thresholding is known to be problematic in such cases, we implemented the change point detection (CPD) method to quantify the statistically-significant emission intensities and corresponding temporal durations exhibited by each emitter^38–40,57,58^. Indeed, for the 5ROX and R6G molecules shown in Fig. 2A, B, CPD analysis reveals 5 and 7 distinct intensities, respectively.

In addition to quantifying the number of distinct intensities within a trace, CPD also parses blinking events into various types: emissive (on), non-emissive (off), segments, and intervals. A segment refers to an event at a particular intensity, whereas an interval is one or more successive segments that occur prior to a switch between on and off. Each of these event types may be useful for BBM, to understand the blinking mechanism, and to determine the origin(s) of multiplexing. For example, interval durations relate to the dark-state production/decay mechanism (i.e., on-off switching) and segment durations report on dynamic changes to the ground- and excited-state properties (e.g., on-on switching due to spectral diffusion)^50,59^. Single-molecule emission intensity can also report on spectral diffusion, local environmental effects (e.g., pH, polarity), as well as conformational flexibility^41,47,59^. Therefore, we used 10 independent statistics output by CPD to quantify and interpret blinking: the number of distinct intensities (*N*_I_), minimum and maximum emissive intensities (*I*_*min*_ and *I*_*max*_), time-averaged intensity (${\langle I{{\rangle }}}_{t}$), average duration of on and off segments (〈*t*_*on,seg*_〉 and 〈*t*_*off,seg*_〉), average duration of on and off intervals (〈*t*_*on,int*_〉 and 〈*t*_*off,int*_〉), and the number of on and off segments in each trace (*N*_*on,seg*_ and *N*_*off,seg*_).

In addition to revealing dynamic heterogeneity of individual molecules (e.g., by the temporal fluctuations in emissive intensity present in Fig. 2B), CPD analysis demonstrates how blinking statistics vary among molecules within a class as well as from class to class. Table S1 in the Supplementary Information presents the average blinking statistics obtained from CPD analysis of many molecules of 5ROX (*n* = 95), R123 (*n* = 132), R560 (*n* = 64), R6G (*n* = 148), and RB (*n* = 71). The blinking statistics of each rhodamine class are quite dispersed, as evidenced by large relative standard deviations and associated distributions spanning several decades in time, intensity, or number of events (Figures S2-S7). These observations are consistent with prior reports^38,39,56^, indicating that: (1) blinking measurements probe a representative population of molecules, (2) individual blinking statistics alone cannot classify emitters as expected^13^, and (3) the rhodamines are a good model system to investigate the molecular limits of BBM.

Indeed, the average CPD-derived blinking statistics of 5ROX, R123, R560, R6G, and RB are generally equivalent within error, consistent with their reported dispersive ET behavior^38,39^. In some cases, however, statistically-significant differences in average blinking statistics are observed. For example, the average *N*_*I*_, *I*_*min*_, 〈*I*〉_*t*_, *N*_*on,seg*_, and *N*_*off,seg*_ values are smaller for 5ROX as compared to R6G (Table S1). For the specific 5ROX and R6G molecules shown in Fig. 2, 〈*I*〉_*t*_ is equal to 2.9 and 6.9 counts, respectively, consistent with the observation that the 5ROX molecule appears to undergo irreversible photobleaching relatively early in the trace, thereby reducing its time-averaged emission intensity. Interestingly, the average ${I}_{max }$ values for 5ROX and R6G are equivalent within error, inconsistent with their ensemble-averaged brightness in solution (i.e., reported ${{\varepsilon }_{532}\phi }_{f}$ values are approximately 40,000 and 100,000 M^-1^ cm^-1^, respectively)^35–37^. This observation can be attributed to modifications in brightness on glass relative to solution and/or photoexcitation of a subpopulation of 5ROX molecules possessing a ${\phi }_{f}$ greater than that of the ensemble average. Collectively, CPD analysis indicates that while the blinking dynamics of 5ROX, R123, R560, R6G, and RB appear quite similar, consistent with a shared on-off switching mechanism, some statistics may yet be useful for BBM.

To investigate further, we used multinomial logistic regression (MLR), a machine learning technique for binary or multi-classification problems that we previously implemented for BBM^14^. MLR works by using the set of 10 CPD-derived blinking statistics of each molecule (e.g., *N*_I_, *I*_*min*_, 〈*I*〉_*t*_, 〈*t*_*on,seg*_〉) and its associated class (e.g., 5ROX, R123, R560) as input predictors. Next, the probability of being class A (*P*_*A*_) is modeled as a logistic (sigmoid) function applied to the normalized blinking statistics according to:

1 $${P}_{A}=\frac{{e}^{({m}_{A}{N}_{I}+{n}_{A}{I}_{min }+\ldots {b}_{A})}}{{\sum }_{j=1}^{K}{e}^{({m}_{j}{N}_{I}+{n}_{j}{I}_{min }+\ldots {b}_{j})}}$$

The resulting fit parameters are a linear combination of regression coefficients (*m*_*j*_*, n*_*j*_, …) and an intercept (*b*_*j*_) associated with each input predictor (*N*_I_, *I*_*min*_,…) and the *j*^th^ outcome. Thus, MLR yields two valuable outputs that can be used to both quantify BBM performance and elucidate its physical origin(s). First, MLR produces fit parameters that are unique to each classification problem and report on the blinking statistics that govern classification. Second, once the fit parameters are determined for a known dataset, the algorithm produces probability outputs (i.e., *P*_*A*_ values) for unknown molecules that can be exploited to maximize performance for a particular application.

When BBM via MLR is applied to the binary classification of all 243 5ROX and R6G molecules, the resulting best fit to a logistic function corresponds to:

2 $$	{-2.7I}_{min }{{-0.8{N}_{{off},{seg}}{-0.7\,\left\langle I\right\rangle }_{t}+0.6N}_{{on},{seg}}+0.4\,\left\langle {t}_{{on},{{int}}}\right\rangle +0.4I_{max }}\\ 	-0.4{N}_{I}-0.3\,\left\langle {t}_{{on},{seg}}\right\rangle + 0.2\,\left\langle {t}_{{off},{{{int}}}}\right\rangle -0.2\,\left\langle {t}_{{off},{seg}}\right\rangle -1.3$$

Equation 2 demonstrates the regression coefficients corresponding to ${I}_{min ,}{N}_{{off},{seg}},{\left\langle I\right\rangle }_{t},$ and *N*_*on,seg*_ have the largest relative magnitude (i.e., -2.7, -0.8, -0.7, 0.6, respectively) and therefore are most important for classification. Figure 2C shows the corresponding confusion matrix of true versus predicted outcomes for 243 5ROX and R6G molecules. When the default classification threshold is applied (i.e., *P*_A_ > 0.5 is automatically classified as emitter A), a minimum classification accuracy of 86.4% is observed, corresponding to a high true positive rate (TPR) of 0.86 and modest false positive rate (FPR) of 0.13. However, prior studies have shown that the classification threshold can be increased to maximize model accuracy^14,60,61^. Figure 2D demonstrates this principle for 5ROX versus R6G. If the classification threshold is raised from 0.5 and “less certain” molecules are increasingly discarded from the dataset, then more molecules are accurately classified at the expense of some data loss. The fraction of emitters that remains in the dataset after thresholding is termed data retention^14^. For example, BBM via MLR achieves 90% classification accuracy for 5ROX versus R6G using a threshold of 0.66, while maintaining 88% of the data (i.e., 12% of the 5ROX/R6G data corresponding to 0.5 < *P*_A_ < 0.66 is discarded to yield an 88% data retention). These results demonstrate that accurate two-color BBM of spectrally-overlapped rhodamine dyes is indeed feasible.

We applied the approach summarized in Fig. 2 to the remaining pairs of rhodamine dyes, the results of which are summarized in Table 1. Corresponding MLR fit parameters are presented in Table S2. Throughout this manuscript, we term binary classification problems of class A (positive) versus class B (negative) as “A/B,” ternary classifications of class A (positive) versus class B (negative) versus class C (pivot) as “A/B/C” and so on. The minimum binary classification accuracies range from 86.4% for 5ROX/R6G down to 58.5% for R560/RB, where random guessing corresponds to 50% accuracy. As expected, RB and R560 are not well differentiated using BBM, consistent with RB undergoing photoinduced N-dealkylation to form R560 and associated intermediates on the timescale of blinking measurements. Indeed, even when the threshold is increased above 0.5, the classification accuracy is plateaued at ~60% (see Figure S8 in the Supplementary Information), indicating that the RB population contains a large proportion of R560 photoproducts and/or mono-, di-, and tri-alkylated intermediates^54^. It is likely that photoinduced N-dealkylation also occurs for the other N-substituted rhodamines^42^, 5ROX and R6G, an effect that is likely to impact BBM.

**Table 1:** Binary BBM-based classification results for 5ROX, R123, R560, R6G, and RB.

Classification	minimum			for 93% accuracy	
	accuracy (%)	TPR	FPR	threshold	data retention (%)
5ROX/R6G	86.4	0.86	0.13	0.97	26
R6G/RB	80.4	0.83	0.31	0.95	28
5ROX/R560	76.1	0.85	0.38	0.92	35
R123/RB	69.5	0.83	0.55	0.87	22
R6G/R123	81.8	0.8	0.17	✗	
R6G/R560	71.2	0.87	0.66	✗	
R123/R560	69.9	0.86	0.63	✗	
5ROX/RB	69.3	0.78	0.42	✗	
5ROX/R123	64.3	0.34	0.14	✗	
R560/RB	58.5	0.47	0.31	✗	

Whereas BBM cannot distinguish RB from R560 as expected, 6 of the 9 remaining rhodamine pairs reach 90% classification accuracy with moderate data retention (i.e., retention ranges from >75% down to ~25%). Furthermore, four of the rhodamine pairs achieve at least 93% classification accuracy. Table 1 shows that 5ROX/R6G, R6G/RB, 5ROX/R560, and R123/RB are the best pairs for BBM in terms of highest minimum classification accuracy (when all the blinking data is included) as well as those that reach 93% accuracy via thresholding, though corresponding data retention is modest at ~25%. To validate MLR-based classification, we performed additional analyses and control experiments (see Figure S9 in the Supplementary Information). First, to determine if classification accuracy is artificially high for the smallest datasets, we performed MLR analyses as a function of the number of molecules (*n*) included in training and testing. When datasets are too small (i.e., *n* $\le$ 10), classification accuracy is poor and the expected minimum 50% accuracy, which is no better than random guessing, is observed. However, at sufficiently high *n* values of $\ge$ 50, both classification accuracy and the relative magnitude of the MLR coefficients are observed to stabilize with further increases to *n*. These results confirm the importance of sampling considerations when using MLR for BBM, while also indicating that the classification results are unrelated to sample size. In addition, by performing additional blinking measurements on mixed samples of known composition, we demonstrate that BBM yields predictions that are in agreement with expected values (Figure S9). Overall, these findings confirm that accurate two-color BBM is achieved with several of the rhodamine dyes included in this study, despite their complex blinking dynamics, broad photophysical distributions, and shared on-off switching mechanism. By correlating the BBM performance of these fluorophores to their molecular structures, we next examine the physical origin(s) of blinking and this somewhat surprising multiplexing capability.

### Impact of rhodamine structure, spectral properties, and dispersive ET on BBM

To determine how structural variations among 5ROX, R123, R560, R6G, and RB manifest in blinking and corresponding BBM performance, we examined the pairs that exhibit the highest classification accuracies (i.e., 5ROX/R6G, R6G/RB, 5ROX/R560, and R123/RB) alongside their MLR fit parameters and CPD-derived blinking statistics. For these sets, MLR analysis reveals that ${I}_{min ,}{{N}_{I},N}_{{off},{seg}},\langle{t}_{{off},{seg}}\rangle$ and $\langle{t}_{{off},{{{int}}}}\rangle$ are generally the most important statistics for classification (Table S2). Whereas *I*_*min*_ and *N*_*I*_ report on brightness and fluctuations in emissive intensity (i.e., on-on switching), the latter statistics relate to the number and duration of excursions to the dark state. How do these statistics connect to the structural and corresponding photo-physics and -chemistry of 5ROX, R123, R560, R6G, and RB? For 5ROX/R6G, 5ROX/R560, R6G/RB, and R123/RB, either ${I}_{min }$ (i.e., the minimum emissive intensity) or ${N}_{I}$ (i.e., the number of distinct intensities) is the most important statistic for classification even though these distributions are considerably broad and overlapped (Figure S2-S7), further highlighting the benefit of MLR-based classification over the individual blinking statistics. One possibility is that differences in ${I}_{min }$ among the rhodamines are simply related to their brightness. For example, photoexcitation of 5ROX, R123, and R560 at 532 nm is expected to generate less absorption as compared to that of R6G and RB (Figure S1). However, MLR analysis shows that *I*_*max*_ is relatively unimportant for classification, indicating that brightness alone does not govern classification. Rather, the generation of “dim” emissive states during blinking seems to be a critical differentiating factor.

Prior studies have reported that photoinduced N-dealkyation^42,54,55^, conformational flexibility^41,62^, and other environmental effects (e.g., pH, polarity)^32,35,63^ can influence the spectral properties and corresponding emission intensity of rhodamines. A recent single-molecule study by Zhang et al. demonstrated that a close structural analog of 5ROX [i.e., rhodamine 101 (R101)] as well as R560, RB, and TAMRA dyes exhibit appreciable spectral shifts and corresponding intensity fluctuations on glass substrates^41^. The static and dynamic variations of R101, RB, and TAMRA were attributed to rotational flexibility of their N,N-dialkylated groups and, to a lesser extent, spectral diffusion associated with N-dealkylation. The same study also showed that neither polarity sensitivity nor the formation of a UV-absorbing lactone^35^ are responsible for the spectral heterogeneity of RB on glass at 532 nm. Therefore, the differences in dim state production and associated ${I}_{min }$ and ${N}_{I}$ values for 5ROX, R123, R560, R6G, and RB are most likely related to their brightness, rotational flexibility, and susceptibility to N-dealkylation. That is, BBM can leverage spectral properties and associated diffusion for classification. Based on their structures, we can collect these rhodamines into three groups—molecules that can populate dim states through both N-dealkylation and rotation about their N,N-dialkylated groups (i.e., R6G and RB), only dealkylation (i.e., 5ROX), and neither effect (i.e., R123 and R560). These structural differences can help to rationalize some of the BBM results (e.g, high accuracy for 5ROX/R6G, 5ROX/R560, and R123/RB), but not others. For example, within this framework, why are R6G and RB are differentiable using BBM?

In addition to spectral diffusion due to conformational flexibility and/or photochemistry, other processes that contribute to the emission-time traces are likely operative. First, the observed intensity fluctuations of R560, as evidenced by relatively high ${N}_{I}$, ${N}_{{on},{seg}}$, and ${N}_{{off},{seg}}$ values, cannot be attributed to the production of dim rotational conformers or dealkylated photoproducts. Therefore, we cannot exclude the possibility that lactone formation (i.e., for 5ROX, R560, and RB) as well as static and dynamic variations in local environment (e.g., polarity, pH) contribute to blinking and BBM performance. The on-off switching dynamics of 5ROX, R123, R560, R6G, and RB also play a major role in blinking. Indeed, MLR analysis reveals that the duration of dark-state excursions is important for R6G/RB and R123/RB classifications. To test the hypothesis that the kinetics of dark-state production and decay for 5ROX, R123, R560, R6G, and RB contribute to BBM performance, we examined their on-off switching mechanism more closely.

The functional form and fit parameters of the on- and off-interval duration distributions are commonly used to report on the physical mechanism responsible for population and depopulation of the dark state. For example, if blinking occurs through a low-lying triplet state, the on- and off-event durations are fit to exponential functions to extract the rate constants for intersystem crossing and decay to the singlet ground state, consistent with first-order kinetics. However, in many blinking studies, the event distributions do not follow simple exponential functions because the underlying mechanism responsible for blinking is not first-order^11^. Prior single-molecule studies of rhodamine dyes immobilized on glass and TiO_2_ substrates have reported lognormal distributions of on and off intervals^38–40^, consistent with a dispersive kinetics model first proposed by Albery wherein the activation barriers to ET are normally distributed^64^. Many other blinking studies have reported power-law distributions, whose origin has and continues to be interpreted in a number of ways such as ET via tunneling, a charging model, and multiple recombination centers^11^.

**Fig. 3:**
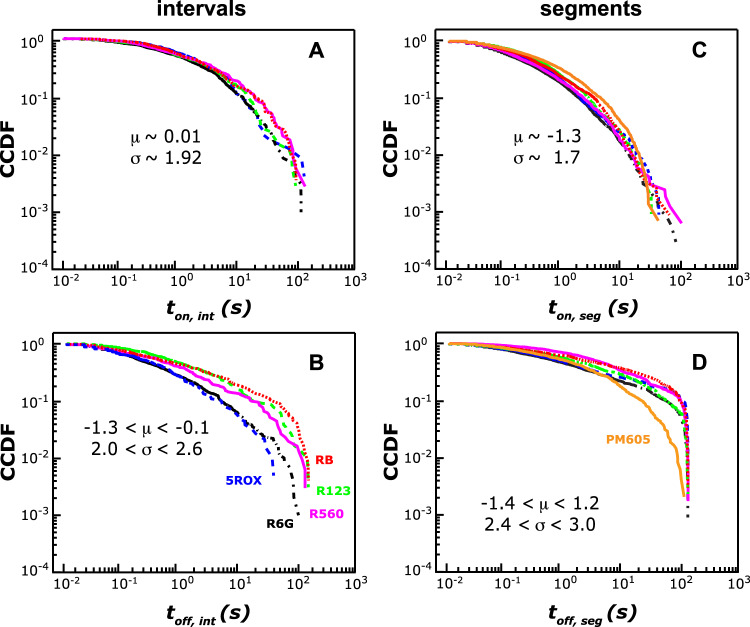
Distributions of blinking event durations for 5ROX, R123, R560, R6G, RB, and PM605. CCDFs of the **A** on-interval and **B** off-interval durations for (blue, dashed) 95 5ROX, (green, dashed) 132 R123, (pink) 64 R560, (black, dash-dotted) 148 R6G, and (red, dotted) 71 RB molecules and immobilized on glass, presented with best-fit parameters to lognormal functions. Although the on-interval durations are considerably overlapped, statistically-significant differences in ${\mu }_{{off}}$ and ${\sigma }_{{off}}$ are observed that support BBM. Corresponding CCDFs of **C** on-segment and **D** off-segment durations for the rhodamines and (orange) 117 PM605 molecules reveal significant overlap among the rhodamines, but the off-segment durations and associated fit parameters of PM605 are distinctive.

To examine the mechanism responsible for on-off switching, consistent with prior treatments, the on- and off-interval durations from many 5ROX, R123, R560, R6G, and RB molecules are converted into complementary cumulative distribution functions (CCDFs) that describe the probability of an event occurring in a time greater than or equal to time, *t*^38,65^. Next, maximum likelihood estimation (MLE) and Kolmogorov−Smirnov (KS) tests are used to quantify best-fit parameters and goodness-of-fits to various test functions. The results of MLE/KS analysis of the on- and off-interval duration CCDFs of 5ROX, R123, R560, R6G, and RB are shown in Fig. 3. In all cases, the distributions are broad, spanning 4 or more orders of magnitude in time, heavy tailed, and best represented by lognormal distributions as compared to other test functions (see Table S3 in the Supplementary Information). These observations are consistent with the Albery model for dispersive ET as expected^38,39^. The on-interval CCDFs are best fit to lognormal distributions corresponding to ${\mu }_{{on}}$ ~ 0.01 and ${\sigma }_{{on}}$ ~ 1.9, where the fit parameters relate to the median and standard deviation of the distribution, respectively. The corresponding off-interval CCDFs are best fit to lognormal functions with varied fit parameters.

The ${\mu }_{{off}}$ values for 5ROX, R6G, R560, RB, and R123 are -1.3, -1.27, -0.7, -0.3, and -0.1, respectively. Corresponding ${\sigma }_{{off}}$ values range from 2.0 to 2.6. Within the Albery dispersive ET kinetics model, ${-\mu }_{{on}}$ and ${-\mu }_{{off}}$ are proportional to the average rate constants for forward and back ET, respectively^39,40^. Therefore, 5ROX, R123, R560, R6G, and RB on glass undergo similar ET kinetics to trap states in glass, but average recombination kinetics decrease as follows: 5ROX ~ R6G > R560 > RB > R123. The $\sigma$ values report on the extent of energetic (and kinetic) dispersion about the mean activation barrier to ET. Therefore, dispersion in the recombination process also seems to be a differentiating factor among the rhodamines, with 5ROX exhibiting the least and RB the most dispersion. The observation that RB exhibits the highest relative dispersion in recombination kinetics (i.e., ${\sigma }_{{off}}$ = 2.6) is consistent with heterogeneity associated with RB as well as its dealkylated photoproduct(s).

This analysis suggests that in addition to emission intensity, differences in the off-interval durations that arise from variations in back ET support BBM. Indeed, 5ROX/R6G, R6G/RB, 5ROX/R560, and R123/RB exhibit some of the largest differences in lognormal fit parameters (Table S3). This is especially evident for R6G (i.e., ${\mu }_{{off}}$ = -1.27 and ${\sigma }_{{off}}$ = 2.07) and RB (i.e., ${\mu }_{{off}}$ = -0.3 and ${\sigma }_{{off}}$ = 2.6), consistent with the finding that $\langle{t}_{{off},{{{int}}}}\rangle$ is the second most important statistic for R6G/RB classification. Collectively, MLR and MLE/KS analyses suggest that differences in spectral properties as well as ET kinetics enable accurate classification between rhodamines. The latter is particularly important for R6G/RB classification. For the remaining pairs of rhodamines whose blinking dynamics are relatively indistinguishable, it is likely that their spectral and/or ET properties (i.e., average values and/or associated dispersion) yield significantly-overlapped blinking distributions that not even MLR can parse for BBM.

Although two-color BBM among rhodamines is possible, differences in the blinking behavior of 5ROX, R123, R560, R6G, and RB are relatively modest, as evidenced by the largely overlapped blinking statistics and distributions (Table S1, Figure S2-S7, and Fig. 3). Indeed, when we extended BBM to ternary classification among three rhodamines, the minimum accuracy significantly drops to an average value of 62%. Even with classification thresholding, only one set of rhodamines reaches a 90% accuracy (i.e., for 5ROX/R6G/RB using a threshold of 0.9 and data retention of just 15.3%). Altogether, these results show that two-color BBM with 90% and 93% accuracy is achieved in six and four of the tested rhodamine pairs, respectively, despite their shared on-off switching mechanism. By performing CPD, MLR, and MLE/KS analyses, we find that BBM leverages both spectral features and ET kinetics for their classification. The best performing pairs (i.e., 5ROX/R6G, R6G/RB, 5ROX/R560, and R123/RB) exhibit significant differences in back ET kinetics as well as their propensity to undergo spectral diffusion via rotational flexibility and/or N-dealkylation. However, that BBM requires significant classification thresholding and does not readily extend to ternary classification of these model rhodamines highlights the need to examine probes that exhibit more distinct blinking behavior. Indeed, two-color BBM with 93% accuracy requires that ~75% of the blinking data is discarded.

### Tuning molecular class and blinking mechanism for BBM

Binary BBM of a series of rhodamine dyes demonstrates that, despite sharing a dispersive on-off switching mechanism, subtle structural variations can generate blinking variations that are differentiable using MLR. However, these results also reveal potential limitations of BBM—without significant differences in blinking dynamics, ternary (or higher) classification is challenging. To test the hypothesis that additional structural and/or photophysical differences will generate more distinctive blinking and thereby enhance BBM, we turned to different classes of emitters (i.e., BODIPY, anthraquinone, quantum dots). Owing to their excellent photophysical properties and small size, BODIPY fluorophores are increasingly used for single-molecule and super-resolved imaging^31,43–49^. However, relatively little is reported about their blinking mechanisms. Zimmerman and co-workers showed that a furan-ring-fused BODIPY derivative exhibits minimal blinking as compared to BODIPY 650^48^. Another study showed the average intensity and photostability of BODIPY-FL molecules is enhanced by using an imaging buffer^49^. However, inhomogeneities among molecules were not assessed in these studies and the blinking mechanism not determined. In a separate study of a carboxylic-acid-bearing BODIPY derivative on TiO_2_, the on and off durations are reported to follow power laws, consistent with a diffusion-controlled ET model for blinking^46^. Yet, previous efforts to study BODIPY at the single-molecule level are complicated by the use of intensity thresholding and least-squares fitting to analyze blinking, both of which introduce systematic errors and assume, rather than determine, the underlying photophysics^38,59^.

We examined the BODIPY fluorophore pyrromethene 605 (PM605) in order to characterize its blinking as well as for potential application to BBM. PM605 is a commercially-available probe that exhibits a primary absorption maximum at 544 nm and fluorescence at 565 nm in ethanol (Fig. 1 and Figure S1), the latter of which is overlapped with the emission of 5ROX, R123, R560, R6G and RB. The blinking dynamics of 116 PM605 molecules were measured under the same experimental conditions and quantified using CPD. Table S1 and Figure S6 shows the average CPD-derived blinking statistics and associated distributions of PM605 are generally equivalent within error to those of the rhodamines, except for $\left\langle {t}_{{off},{seg}}\right\rangle$ and to a lesser extent, ${I}_{min }$ and $\left\langle {t}_{{off},{{{int}}}}\right\rangle$. The corresponding on- and off-interval duration CCDFs of PM605 on glass are best fit to lognormal distributions. Surprisingly, the lognormal fit parameters are comparable to some of the rhodamines (i.e., *μ*_*on*_ = 0.3, *σ*_*on*_ = 2.01 and *μ*_*off*_ = -0.7, *σ*_*off*_ = 2.04, Table S3). These results suggest that dispersive ET to/from trap states on glass, or an analogous Albery-type mechanism^40,64^, is responsible for the on-off switching of PM605 as well. A thorough study of the blinking mechanism(s) of PM605 and its structural analogs are underway, though beyond the scope of the present study. For the purpose of BBM, we examined how PM605 performs in two- and three-“color” classification, excluding the set containing both RB and R560.

Table 2 presents the results of binary BBM on PM605 versus 5ROX, R123, R560, R6G, and RB. In all five tested pairs, BBM via MLR yields minimum classification accuracies >75%, and 93% accuracy is achieved using varying degrees of data retention. That is, with the possible exception of PM605/R6G, which achieves 93% accuracy at the expense of 88% data loss, BBM performance is significantly improved for PM605/rhodamine as compared to classification between rhodamines. To understand the structural and mechanistic origin(s) of this result, we examined the MLR fit parameters and blinking statistics of PM605 versus 5ROX, R123, R560, R6G, or RB. The regression coefficients from MLR analysis (Table S4) demonstrate that ${I}_{min }$ and $\left\langle {t}_{{off},{seg}}\right\rangle$ are the most important statistics for PM605/rhodamine classification. The significance of *I*_*min*_ is consistent with the production of more dim states in 5ROX, R123, R560, R6G, and RB as compared to PM605, the latter of which cannot undergo N-dealkylation or lactone formation and is unlikely to form dim rotational conformers^47^. Indeed, average *I*_*min*_ is higher for PM605 relative to the rhodamines (Table S1). The importance of $\left\langle {t}_{{off},{seg}}\right\rangle$ indicates that the durations of individual excursions to the dark state are somewhat distinct for PM605 as compared to the rhodamines. Figure 3C, D present the on- and off-segment CCDFs for PM605, 5ROX, R123, R560, R6G, and RB. The on-segment CCDFs are significantly overlapped, but the corresponding off-segment distributions and associated fit parameters (Table S5) of PM605 are distinctive. Altogether, MLR analysis demonstrates that PM605 undergoes sufficiently distinct blinking to enhance BBM relative to the rhodamines alone. This observation highlights the need for future mechanistic studies (i.e., to elucidate the origin of on-off and on-on switching) of PM605 and its structural analogs.

**Table 2:** Binary BBM-based classification results with PM605, AZ, and the rhodamines.

Classification	minimum			for 93% accuracy	
	accuracy (%)	TPR	FPR	threshold	data retention (%)
5ROX/PM605	85.3	0.84	0.14	0.78	79
PM605/R123	82.7	0.81	0.16	0.93	40
PM605/RB	82.4	0.84	0.20	0.82	73
PM605/R560	78.9	0.83	0.28	0.88	52
PM605/R6G	76.1	0.70	0.19	0.95	12
5ROX/AZ	97.9	0.97	0.01	✓	
AZ/RB	94.5	0.96	0.08	✓	
AZ/R123	92.8	0.94	0.08	✓	
AZ/R560	87.6	0.92	0.22	0.83	77
AZ/R6G	82.3	0.82	0.17	0.79	75
AZ/PM605	74.4	0.81	0.34	0.88	29

When PM605 is introduced into ternary classification problems, the average minimum accuracy is increased from 62% for three rhodamines to 69.5% for two rhodamines and PM605 (Table S6). The best BBM performance is observed for 5ROX/PM605/RB, which exhibits a minimum accuracy of 70.9%. Using a threshold of 0.8, a 90% classification accuracy is achieved for this set, with a data retention of ~50%. Altogether, 90% classification accuracy can be achieved for 4 of the 9 sets of PM605 versus two rhodamines (i.e., 5ROX/PM605/RB, 5ROX/PM605/R560, PM605/R123/RB, and PM605/R123/R560), though the corresponding average data retention is low at 35%. These results represent the first successful classification of three spectrally-indistinct fluorophores based solely on their blinking. The inclusion of PM605 improves BBM performance relative to the rhodamines alone, as evidenced by extending BBM to three colors as well as enhancements in minimum accuracy, data retention, and the number of sets that reach $\ge$90% accuracy. However, the blinking behavior of PM605 is unexpectedly alike that of the rhodamines in the same experimental conditions, indicating that BBM performance can be further enhanced by implementing more drastic structural and mechanistic changes.

To determine if more significant photophysical changes generate further enhancements to BBM, we turned to a class of fluorophores that are known to undergo a different blinking mechanism. We previously examined several anthraquinone dyes including 1,2-dihydroxyanthraquinone [alizarin (AZ)], a molecule that exhibits broad emission from ~535 to 700 nm (Fig. 1) due to an excited-state intramolecular proton transfer (ESIPT) process to form an emissive phototautomer^50^. By studying its blinking dynamics on glass at 532 nm, we showed that ESIPT generates a large number of on segments as well as decreases the likelihood of AZ to undergo ET and subsequent photobleaching. Therefore, BBM-based classification with AZ should be more accurate relative to the rhodamines and PM605. The average CPD-derived blinking statistics and corresponding distributions of 146 AZ molecules on glass are shown in Table S1 and Figure S7. Although the average blinking statistics of AZ are not remarkably different as compared to the other molecules included in this study, the distributions of on- and off-intervals are quite distinct. Tan et al. showed the on intervals of AZ are lognormally distributed (i.e., *μ*_*on*_ = 0.8, *σ*_*on*_ = 1.6) and off intervals follow a Weibull distribution (i.e., the product of a stretched exponential and fractional power law)^50^. That the functional form and fit parameters of these distributions are significantly different for AZ relative to 5ROX, R123, R560, R6G, RB, and PM605 (Tables S4) is consistent with ESIPT being operative.

Table 2 shows that BBM performance of AZ versus 5ROX, R123, R560, R6G, or RB is exceptional. The minimum classification accuracies range from a high of 97.9% for AZ/5ROX down to 82.3% for AZ/R6G. All five tested sets achieve $\ge$ 93% accuracy while maintaining a large proportion of the data (i.e., retention ranges from 100% to 75%). The MLR regression coefficients shown in Table S4 demonstrate that *I*_*min*_ and 〈*I*〉_*t*_ are the most important statistics for AZ/rhodamine classification. The significance of *I*_*min*_ is consistent with differences in brightness and dim state production in AZ versus the rhodamines. For example, average *I*_*min*_ is higher for AZ as compared to 5ROX, R123, R560, R6G, and RB (Table S1 and Figures S2-S7), indicating that the phototautomer of AZ exhibits brighter emission relative to the rhodamines’ dim states. Time-averaged intensity (i.e., 〈*I*〉_*t*_) is also important for classification, consistent with the interpretation that ESIPT has a photoprotective effect on AZ, decreasing its likelihood of excited-state ET and subsequent photobleaching^50^. Most AZ molecules undergo photobleaching after 50 s, but without the help of additives or imaging buffers to enhance rhodamine photostability, they generally undergo single-step photobleaching within 50 s^13^. In addition to these factors, *N*_*I*_, *N*_*on,seg*_ as well as the functional form and fit parameters of the on- and off-interval duration CCDFs (Table S3) are quite different for AZ as compared to the rhodamines, which also support BBM. The aforementioned statistics are also important for AZ/PM605 classification, though this set is somewhat less effective for BBM as compared to AZ versus the rhodamines. The origin of this behavior will be investigated in a future mechanistic blinking study of PM605 and its derivatives.

When AZ is included in ternary classification attempts with two rhodamines, the average minimum accuracy is increased to 77% (Table S6). As expected, because the blinking behavior of PM605 and the rhodamines are similar, BBM performance of AZ versus two rhodamines or PM605 and one rhodamine are roughly equivalent. The best performance with AZ is observed for 5ROX/AZ/R560, which exhibits a minimum accuracy of 80.6%. For this set, a threshold of 0.8 yields a 90% accuracy with 67% data retention. These results demonstrate that the addition of AZ, a molecule that undergoes ESIPT, increases BBM performance. Indeed, with thresholding, all 9 of the 9 sets of AZ with two rhodamines achieve 90% classification accuracy, with corresponding data retention ranging from approximately 72% down to 25%. If low data retention values of ~35% are workable, then ternary BBM-based classification with AZ reaches at least 93% accuracy.

**Fig. 4:**
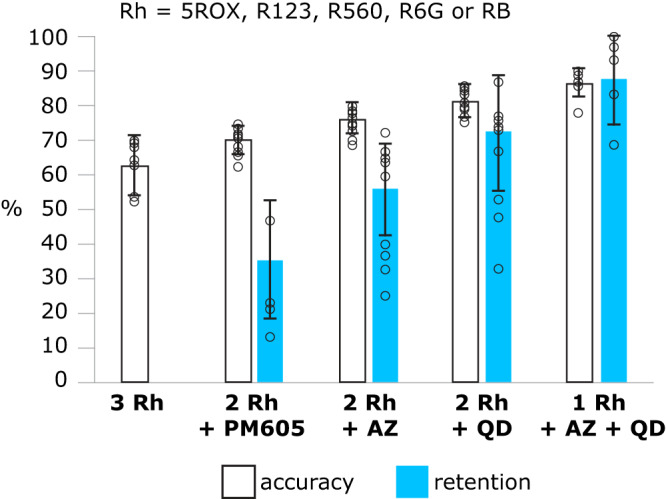
Bar graph summarizing the impact of structure and photophysics on average ternary BBM performance. Error bars, mean $\pm$ s.d. The (black) average minimum classification accuracy is lowest for ternary classification among 3 rhodamines (Rh) and increases as blinking behavior becomes more distinct (from left to right) by incorporating PM605, AZ, and QD emitters. (cyan) Average data retention corresponding to 90% classification accuracy exhibits a concomitant increase. That is, only one classification among 3 Rh reaches 90% accuracy and so average data retention is not reported. As blinking behavior becomes more distinct with PM605, AZ, and then QD, less low-probability data needs to be discarded to achieve 90% accuracy.

Collectively, the results with 5ROX, R123, R560, R6G, RB, PM605, and AZ demonstrate that BBM improves as blinking (i.e., statistics, distributions, on-on and on-off switching mechanisms) becomes more distinct. Figure 4 summarizes how three-color BBM performance is varied with molecular class and corresponding photophysics. The average minimum classification accuracy is increased according to: 3 rhodamines (62.0%), 2 rhodamines with PM605 (69.5%), and 2 rhodamines with AZ (77.0%). Another measure of performance is how many of the tested sets reach at least 90% accuracy and the corresponding data retention. Whereas just one of ternary classifications among rhodamines reach 90% accuracy, four sets with PM605 reach 90% with an average data retention of 35.4%. When AZ is classified against the rhodamines, all nine sets yield 90% accuracy with an average data retention of 56.7%. That is, as differences in blinking between classes are magnified, the quality and applicability of BBM is enhanced and more data can be retained.

To further amplify this point, we included an entirely different class of emitters into the equation. Previous blinking studies have shown that QD exhibit more intensity fluctuations, brighter emission, and improved photostability as compared to R6G^13,14,52,66^. More recently, Snee and coworkers showed that the on- and off-event durations for CdSe/CdZnS dots are well represented by truncated power laws^53^. That is, QD should exhibit significant differences in blinking dynamics and mechanism as compared to the molecular fluorophores included in this study^11^. Indeed, when QD are tested against the fluorophores, the resulting minimum accuracy, number of sets that achieve 90% accuracy, and corresponding data retention are substantially increased. For example, when QD is classified against 2 rhodamines, the average minimum accuracy is 81.1%, and all 9 sets yield 90% accuracy with a corresponding average data retention of 72.0%. For ternary classification of QD versus AZ versus a rhodamine, the average minimum accuracy is further increased to 85.5%. All five tested sets achieve 90% and 93% accuracies with corresponding average data retentions of 87.2% and 73.8%, respectively (Fig. 4). The results of three-color BBM for all sets of emitters included in this study are summarized in Table S6 of the Supplementary Information. Among these 39 trials, 5ROX/AZ/QD has the best BBM performance, likely because it includes emitters that exhibit the most distinct blinking behavior, mechanism(s), and/or photostability^13,50^. For this particular set, a minimum accuracy of 89.2% is observed, which readily yields 93% accuracy with a threshold of 0.6 and corresponding data retention of 90.3%.

The BBM results summarized in Fig. 4 and Table S6 demonstrate accurate three-color BBM among many sets of spectrally-overlapped emitters, including small-molecule probes that would otherwise be difficult to multiplex on the basis of their emission spectra. Of the 39 ternary classification trials, 19 sets of emitters exhibit excellent BBM performance as defined by: (1) a high minimum classification accuracy, and (2) achieving 90% accuracy with data retention of at least 50%. In some cases, high ternary classification accuracy can only be achieved when a disproportionally large number of emitters from one class are discarded via thresholding, effectively reducing a ternary classification problem to a binary one. Therefore, practitioners must be careful during the thresholding process to monitor data retention within each class.

As a final test of the limitations of BBM within the context of the model fluorophores implemented in this study, we increased the complexity of the classification problem to >3 emitters at a time. When BBM is used to multiplex four emitters that exhibit significant differences in blinking (i.e., 2 rhodamines versus AZ versus QD), several sets achieve 90% classification accuracy with moderate data retention. For example, the minimum classification accuracy of 5ROX/AZ/QD/R560 is 77.7%, and an accuracy of 90% is achieved with a threshold of 0.70 and corresponding overall data retention of 65.0%. Importantly, this threshold discards uncertain emitters relatively proportionally across all four classes. However, attempts at five-color classification of the probes included in this study requires that three emitters are from the rhodamine family and/or PM605, all of which exhibit relatively similar blinking behavior. Therefore, as expected, BBM among five of these specific emitters is not particularly effective (i.e., the average minimum classification accuracy is ~65%).

By examining the blinking dynamics and BBM performance of eight different probes from several different classes, this study highlights the power of MLR to both multiplex emitters and reveal fundamental insight about blinking. In doing so, is determined a set of guiding principles to achieve accurate multiplexing of four or more emitters. If blinking dynamics and/or mechanism (which includes both on-on and on-off switching) are sufficiently distinct, then BBM via MLR holds the potential to provide for rapid, accurate multiplexing of >4 emitters based solely on their blinking.

## Conclusion

Despite their complex blinking dynamics and broad photophysical distributions, BBM distinguishes four pairs of rhodamine dyes (i.e., 5ROX/R6G, R6G/RB, 5ROX/R560, and R123/RB) with at least 93% accuracy, though at the expense of up to 75% data loss. That is, BBM does not require probes to have completely different blinking mechanisms. By correlating the BBM performance and blinking statistics of these fluorophores to their structures, we demonstrate that differences in ET kinetics and spectral diffusion properties govern classification. The best performing pairs exhibit significant differences in their back ET kinetics as well as their propensity to undergo spectral diffusion via rotational flexibility and/or N-dealkylation. Therefore, for rhodamine probes that undergo an Albery-type blinking mechanism, BBM performance can be enhanced by selecting fluorophores with different ET kinetics or redox activity, and if possible, controlling dispersion—perhaps through the addition of an imaging buffer^16,49^.

By understanding how structure controls the blinking dynamics of 5ROX, R123, R560, R6G, and RB for BBM, we next sought to amplify differences in blinking and enhance performance by introducing structural and photophysical modifications. To this end, we investigated the blinking dynamics of PM605, AZ, and QD, which undergo different photo-physics and -chemistry. Although PM605 belongs to a different class of fluorophores and is less prone to spectral diffusion, its blinking dynamics are relatively similar to those of the rhodamines, consistent with a dispersive ET mechanism for on-off switching. However, BBM performance for PM605 versus 5ROX, R123, R560, R6G, or RB is improved – in terms of accuracy and data retention –relative to the rhodamines alone. Using CPD, MLR, and MLE/KS analyses we demonstrated that differences in dim state production as well as the dark state lifetime are most important for PM605/rhodamine classification. This means that PM605 and its structural analogs, some of which have been implemented for SMLM^18,43–45^, are viable probes for BBM, provided their dark-state population and decay kinetics can be varied through structural means. Such mechanistic and applied studies are underway. By introducing AZ and QD emitters that are known to undergo different blinking mechanisms (e.g., ESIPT and dispersive charge transfer processes, respectively), we demonstrate two-, three-, and four-color BBM with $\ge$ 93% accuracy. In addition to differences in the on-off switching properties of AZ versus the rhodamines, the ability of AZ to undergo ESIPT enhances its photostability, also promoting classification. Thus, controlling the timescale and mechanism of molecular photobleaching may be a useful parameter for the design and implementation of BBM probes.

Ultimately, this study elucidates the structure-activity relationships governing BBM performance in a series of model fluorophores that exhibit a variety of photo-physical and -chemical processes during blinking. In doing so, accurate two-, three-, and four-color BBM using spectrally overlapped, small-molecule probes is demonstrated, though some data loss via classification thresholding is required. Although these findings provide guidelines for the selection and design of fluorescent probes for BBM, several practical challenges remain to be addressed. For example, demonstrating multiplexed SMLM using BBM will require the training and testing of probes at higher labeling densities (>1 molecule/100 nm^2^) and with emitters that are optimized for their high localization precision and low duty cycle (e.g., spontaneously blinking Si-rhodamines)^16,32,67^. Wiseman et al. developed an image correlation method to extract blinking statistics from SMLM data at high labeling densities^68^, which we plan to implement for this purpose. Since blinking dynamics and their associated distributions are known to broaden in heterogeneous environments, it is also critical to demonstrate BBM in biological systems. We previously investigated two-color BBM between R6G and QD in a model biological environment [i.e., poly(vinyl alcohol) matrix]^69^. Surprisingly, the distributions of several blinking statistics for R6G were broader on glass relative to PVA^14^, suggesting that in some cases BBM might actually improve in biological systems, especially with the use of imaging buffers to control blinking and associated dispersion.

With an understanding of how molecular structure and blinking dynamics govern BBM in a series of model fluorophores, future studies will target further enhancements in accuracy and retention using BODIPY and Si-rhodamine probes in various environments for multicolor SMLM. By exploiting the intrinsic blinking dynamics of single emitters for multiplexing, BBM holds the promise to significantly expand the number of available probes for SMS and SMLM experiments, improving the versatility of these tools for biological and materials investigations.

## Methods

### Materials, sample preparation, bulk characterization

5ROX (Thermo Fisher, 5-carboxy-X-rhodamine, triethylammonium salt, single isomer, >97%), AZ (Acros Organics, 97%), PM605 (Exciton, 8-Acetoxymethyl-2,6-diethyl-1,3,5,7-tetramethyl pyrromethene), QD (Invitrogen, Qdot 565 ITK carboxyl quantum dots, 8 μM solution in borate buffer), R123 (Acros Organics, 99 + %), R560 (Exciton, 99%), R6G (Acros Organics, 99%), and RB (Acros Organics, 99%) were used as received. Nanomolar solutions of the emitters were prepared in base-bathed glassware (KOH, 12–24 h) and briefly sonicated prior to use. 5ROX, QD, R6G, R123, RB, and R560 solutions were prepared in Type I ultrapure water (Thermo Scientific, Easy Pure II, 18.2 MΩ cm). AZ and PM605 solutions were prepared in absolute ethanol (Pharmco-Aaper). Samples for single-molecule microscopy were prepared by base bathing glass coverslips (Fisher Scientific, 12-545-102) for 12–24 h, followed by rinsing with ultrapure water, and drying with air (Wilkerson, X006-02-000). A 35 µL aliquot of ~1 nM emitter solution was spun coat (Laurell Technologies, WS-400-6NPP-LITE) onto the glass coverslip at 3000 rpm for 30 s with a 5 s acceleration time. Corresponding ensemble-averaged absorption and emission spectra of ~10^-5^–10^-6 ^M aqueous and ethanolic solutions of the emitters were measured in 1-cm quartz cuvettes using an UV-Vis (Perkin Elmer Lambda 35) or fluorescence spectrometer (FS5, Edinburgh Instruments), respectively.

### Single-molecule microscopy & blinking analyses

Samples were fastened in a custom aluminum flow cell (~1.5 × 3 × 0.5 in.), flushed with dry N_2_ via Tygon tubing before and throughout each experiment a rate of 0.2–0.5 scfh (Key Instruments, MR3A01AVVT), and set on a nanopositioning stage (Physik Instrumente, LP E-545) atop an inverted confocal microscope (Nikon, TiU). Laser excitation (Spectra Physics, Excelsior, 532 nm) at 0.99 ± 0.05 µW excitation power (i.e., 1.1 kW/cm^2^ at the sample) was focused to a diffraction limited spot utilizing a high numerical aperture (NA) 100× oil-immersion objective (Nikon Plan Fluor, NA = 1.3). Emission from the sample was passed back through the objective, a long-pass filter (Semrock, LP03-532RS-2S), and collected using an avalanche photodiode detector with a 50 μm aperture (MPD, PDM050CTB) to achieve confocal resolution. A z-axis microscope lock (Applied Science Instruments, MFC-2000) was used to minimize focal drift during raster scans. A custom LabView program was used to control the nanopositioning stage and collect emission micrographs using a 30-ms bin time over a ~ 10 × 10 µm^2^ area. Corresponding blinking dynamics of individual diffraction-limited spots (i.e., ~250–350 nm FWHM) were measured using a 10 ms bin time for 150 s for all emitters except AZ. The AZ blinking data is from a previous study that employed a 100-s experimental window^50^, and therefore, all blinking traces to be classified against AZ were truncated to the first 100 s.

The change point detection (CPD) method for binned data, described in detail by Kopera and coworkers, was used to quantify blinking dynamics^40,58^. This approach is based on the method described by Yang and coauthors, which determines the locations of statistically significant intensity change points within a series of individual photon arrival times via a generalized likelihood ratio test^57,58^. Importantly, the CPD method for binned data determines change points using critical values that are determined for a given number of bins and photons. In CPD analysis, the lowest intensity is denoted as off. On events are defined as those with intensities one standard deviation above the rms noise. CPD analysis yields 10 blinking statistics from each trace that are standardized by z-score normalization and then used as input predictors, along with emitter identity, for fitting and classification using multinomial logistic regression (MLR) analysis (Matlab R2023b, mnrfit and mnrval)^14^. For binary classification problems, MLR produces a single set of fit parameters. For ternary classification, there are three classes in Eq. 1 (i.e., *K* = 3), and therefore two sets of regression coefficients with respect to the third or ‘pivot’ class. After the fit parameters are established using MLR, emitter identities are removed, the dataset is split into training (90%) and testing (10%) sets, and 10-fold cross validation is performed to assess the model’s predictions when emitter class is unknown.

### Supplementary information

Supplementary Information

## Data Availability

All data are available from the corresponding author upon reasonable request.

## Code availability

The change point detection method for binned data is available at zenodo.org with 10.5281/zenodo.10471846.
