# Supplementary material for: Blinking characteristics of organic fluorophores for blink-based multiplexing
Source: Commun Chem. 2024 Jan 27;7:18. doi: 10.1038/s42004-024-01106-5 (PMC10821931; doi:10.1038/s42004-024-01106-5)
Supplement: Supplementary file 1 — Supplementary Information [file 42004_2024_1106_MOESM1_ESM.pdf]

Supplementary Information for

Blinking characteristics of organic fluorophores for blink-based multiplexing

*Amelia G. Seabury,[1] Alisha J. Khodabocus,[1] Isabelle M. Kogan,[1] Grayson R. Hoy,[2] Grace A. DeSalvo,[1] Kristin L. Wustholz[1]**

[1]Chemistry Department, William & Mary, Williamsburg, VA, USA
**email: kwustholz@wm.edu*

Additional Tables & Figures:

Figure S1. Ensemble-averaged absorption spectra of all probes (S-2)

Table S1. Average CPD-derived blinking statistics of all emitters included in this study (S-3)

Figure S2. Distributions of CPD-derived blinking statistics of 5ROX versus R6G (S-4)

Figures S3 – S7. Distributions of CPD-derived blinking statistics for R123, R560, RB, PM605, and AZ, respectively. (S-5 to S-9)

Table S2. Best-fit parameters for binary classification of 5 Rh dyes using MLR (S-10)

Figure S8. Binary classification accuracy of RB/R560 (S-11)

Figure S9. Additional MLR analyses as a function of sample size and on mixed samples of known composition (S-12)

Table S3. MLE/KS fitting results of the distributions of on- and off-interval durations for all fluorophores in this study (S-13)

Table S4. Best-fit parameters for binary classification of 1 Rh versus PM605 or AZ using MLR (S-14)

Table S5. MLE/KS fitting results of the on- and off-segment distributions (S-15)

Table S6. Ternary BBM-based classification results for 5ROX, R123, R560, R6G, RB, PM605, AZ, and QD (S-16 to S-17)

Supplementary References (S-18)

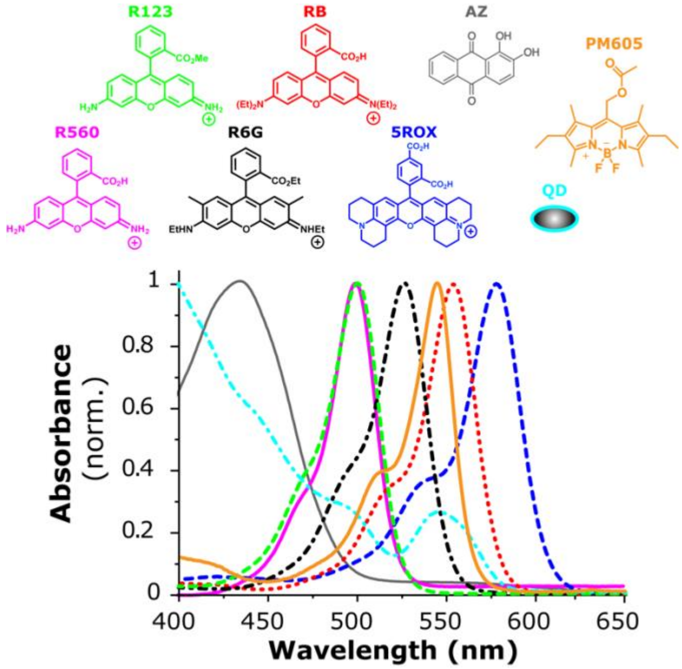

Figure S1. Structures and corresponding ensemble-averaged absorption spectra of the probes examined in this study measured in aqueous or ethanolic solutions: (pink solid) R560, (green dashed) R123, (black dash-dotted) R6G, (red dotted) RB, (blue dashed) 5ROX, (gray solid) AZ, (cyan dash-dotted) QD, and (orange solid) PM605. Primary absorption maxima are observed at 423 nm for AZ, 500 nm for R123 and R560, 526 nm for R6G, 544 nm for PM605, 555 nm for RB, 558 nm for QD, and 578 nm for 5ROX. Previous studies have shown that ethanolic solutions of AZ exhibit a primary absorption maximum at ~425 nm and fluorescence spectra that vary with excitation wavelength and solvent, consistent with the formation of an emissive phototautomer via excited-state intramolecular proton transfer (ESIPT).[1–3] The reported fluorescence maxima of the normal 9,10-keto (N) and 1,10-keto tautomer (T) forms of AZ in ethanol are 530 and 615 nm, respectively.[1,2] At excitation wavelengths longer than ~480 nm, a primary fluorescence peak centered at 524 nm is observed, consistent with emission from the locally-excited N state of the dye. However, previous ensemble-averaged and single-molecule measurements of AZ at 532-nm excitation have reported broad fluorescence from 535 to 700 nm and blinking characteristic of spectral diffusion, which has been attributed to contributions from both the N and T forms of the dye.[1] Shorter excitation wavelengths will further promote ESIPT, which may be useful for BBM.

Table S1. Average CPD-derived blinking statistics for 5ROX (*n* = 95), R123 (*n* = 132), R560 (*n* = 64), R6G (*n* = 148), RB (*n* = 71), PM605 (*n* = 116), AZ (*n* = 146), and QD (*n* = 143) emitters immobilized on glass. Errors correspond to the standard error of the mean. Corresponding standard deviations that measure dispersion are larger by $\sqrt{n}$. The Intensities have units of counts within the 10-ms bin time and durations are in seconds.

Emitter	N_I	$N_{on,seg}$	$N_{off,seg}$	I_{min}	I_{max}	$\langle I \rangle_t$	$\langle t_{on,seg} \rangle$	$\langle t_{off,seg} \rangle$	$\langle t_{on,int} \rangle$	$\langle t_{off,int} \rangle$
5ROX	4.6 ± 0.3	14 ± 3	3.4 ± 0.6	2.7 ± 0.1	35 ± 5	2.8 ± 0.3	1.9 ± 0.3	50 ± 5	8 ± 1	6 ± 2
R6G	5.7 ± 0.3	28 ± 5	7.4 ± 0.8	3.9 ± 0.1	32 ± 3	5.0 ± 0.5	2.4 ± 0.6	32 ± 2	8 ± 1	6 ± 1
R123	4.1 ± 0.3	10 ± 2	4.3 ± 0.4	2.9 ± 0.1	20 ± 3	2.3 ± 0.2	2.1 ± 0.3	39 ± 2	6 ± 1	12 ± 2
R560	7.3 ± 0.5	30 ± 5	6.7 ± 0.9	3.4 ± 0.1	53 ± 8	4.9 ± 0.5	4 ± 2	31 ± 3	12 ± 3	10 ± 2
RB	6.5 ± 0.4	19 ± 3	4.7 ± 0.5	3.1 ± 0.1	51 ± 6	4.3 ± 0.5	1.7 ± 0.3	45 ± 4	8 ± 1	14 ± 2
PM605	5.1 ± 0.3	15 ± 3	4.1 ± 0.6	4.8 ± 0.2	28 ± 3	5.4 ± 0.5	2.8 ± 0.3	14 ± 2	11 ± 2	7 ± 1
AZ	7.2 ± 0.3	18 ± 2	6.6 ± 0.5	6.3 ± 0.5	52 ± 6	7.2 ± 0.9	2.0 ± 0.2	11 ± 1	8 ± 1	8 ± 1
QD	5.7 ± 0.5	210 ± 10	14 ± 1	16 ± 2	130 ± 10	55 ± 5	1.1 ± 0.1	1.9 ± 0.2	13 ± 1	2.8 ± 0.7

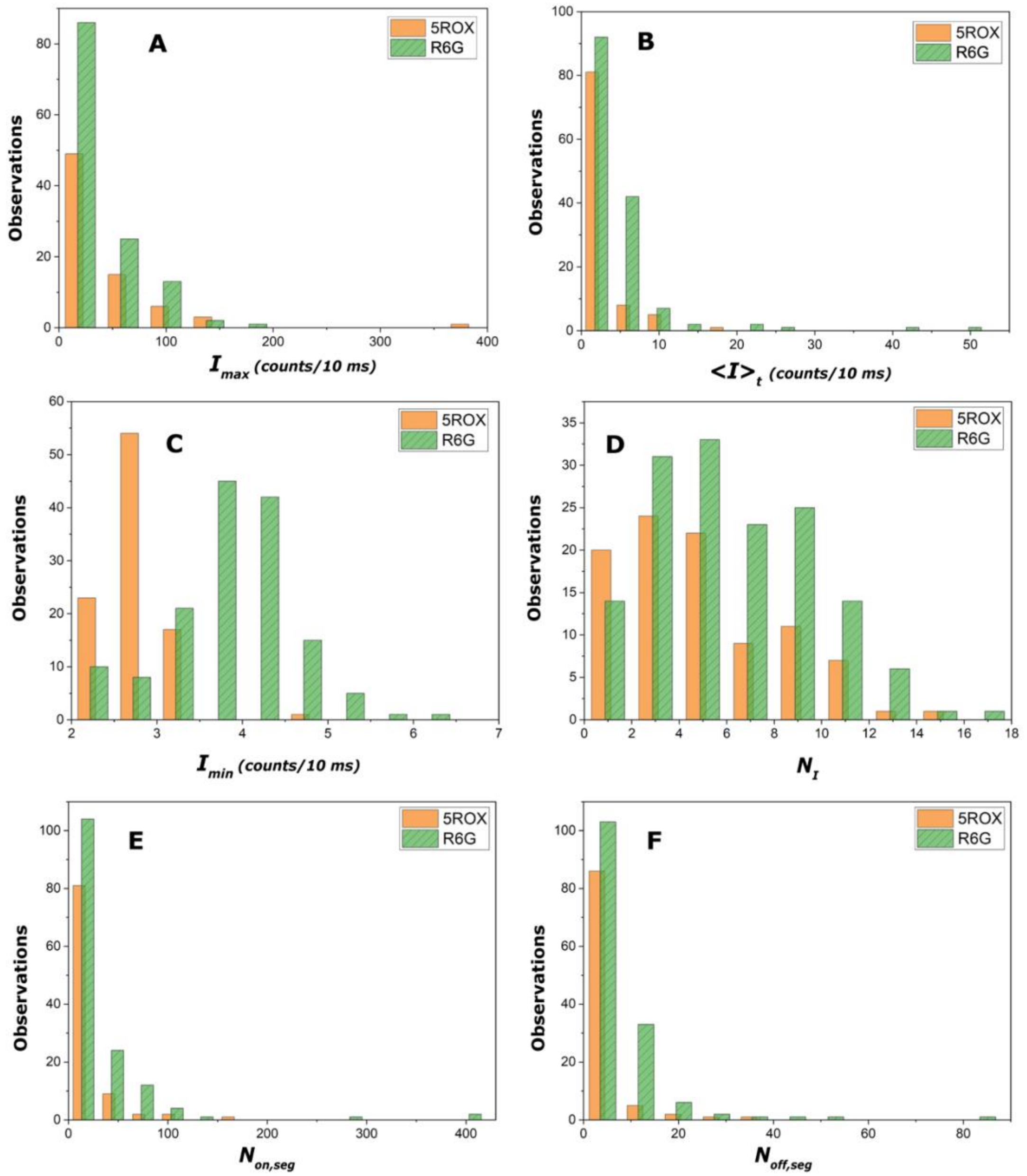

Figure S2. Histograms of (A) I_{max}, (B) $\langle I \rangle_t$, (C) I_{min}, (D) N_I, (E) $N_{on,seg}$, and (F) $N_{off,seg}$ obtained from CPD analysis of (green, dashed) 95 5ROX molecules and (orange, solid) 148 R6G molecules. The distributions are broad and significantly overlapped, highlighting the need to classify using machine learning rather than individual blinking statistics. The corresponding distributions of on- and off-event durations (Figure 3 in the manuscript) are even more dispersive, spanning more than four decades in time.

Figures S3-S7 present histograms of the CPD-derived blinking statistics for all other dye molecules included in this study. The corresponding event duration distributions that are presented in the manuscript (Figure 3) or in Tan et al[1] are more dispersive, spanning more than three or four decades in time. The associated distributions for QD were previously reported by Hoy and coworkers.[4] To facilitate comparison of these distributions between molecules, the bin sizes and ranges of the plots are kept as consistent as possible. The bin sizes in Figures S3-S7 for I_{max}, $\langle I \rangle_t$, I_{min}, N_I, $N_{on,seg}$, and $N_{off,seg}$ correspond to 40 counts 10 ms^{-1}, 2-4 counts 10 ms^{-1}, 0.5-1 counts 10 ms^{-1}, 2, 20, and 4, respectively.

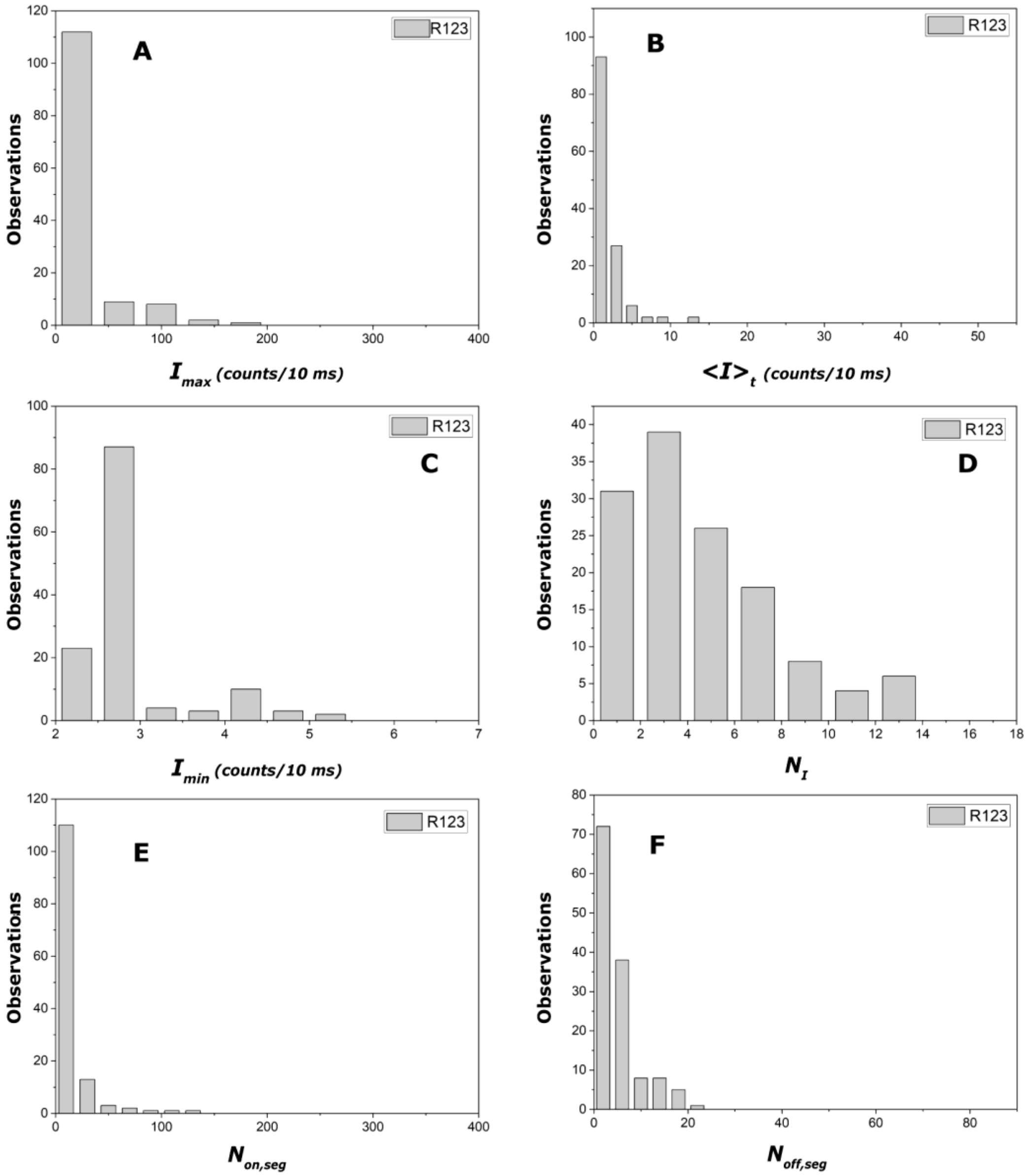

Figure S3. Histograms of (A) I_{max}, (B) $\langle I \rangle_t$, (C) I_{min}, (D) N_I, (E) $N_{on,seg}$, and (F) $N_{off,seg}$ obtained from CPD analysis of 132 R123 molecules.

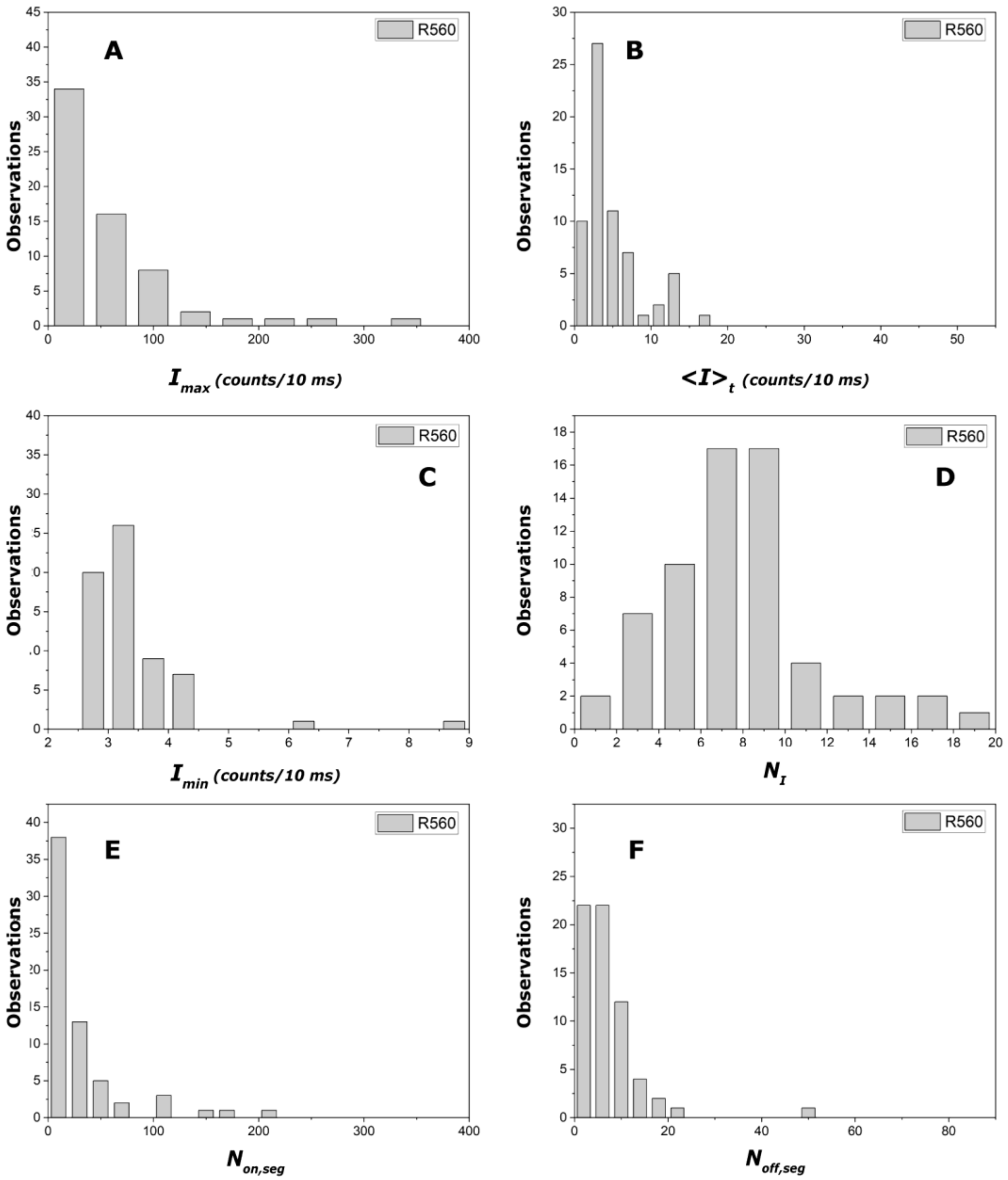

Figure S4. Histograms of (A) I_{max}, (B) $\langle I \rangle_t$, (C) I_{min}, (D) N_I, (E) $N_{on,seg}$, and (F) $N_{off,seg}$ obtained from CPD analysis of 64 R560 molecules.

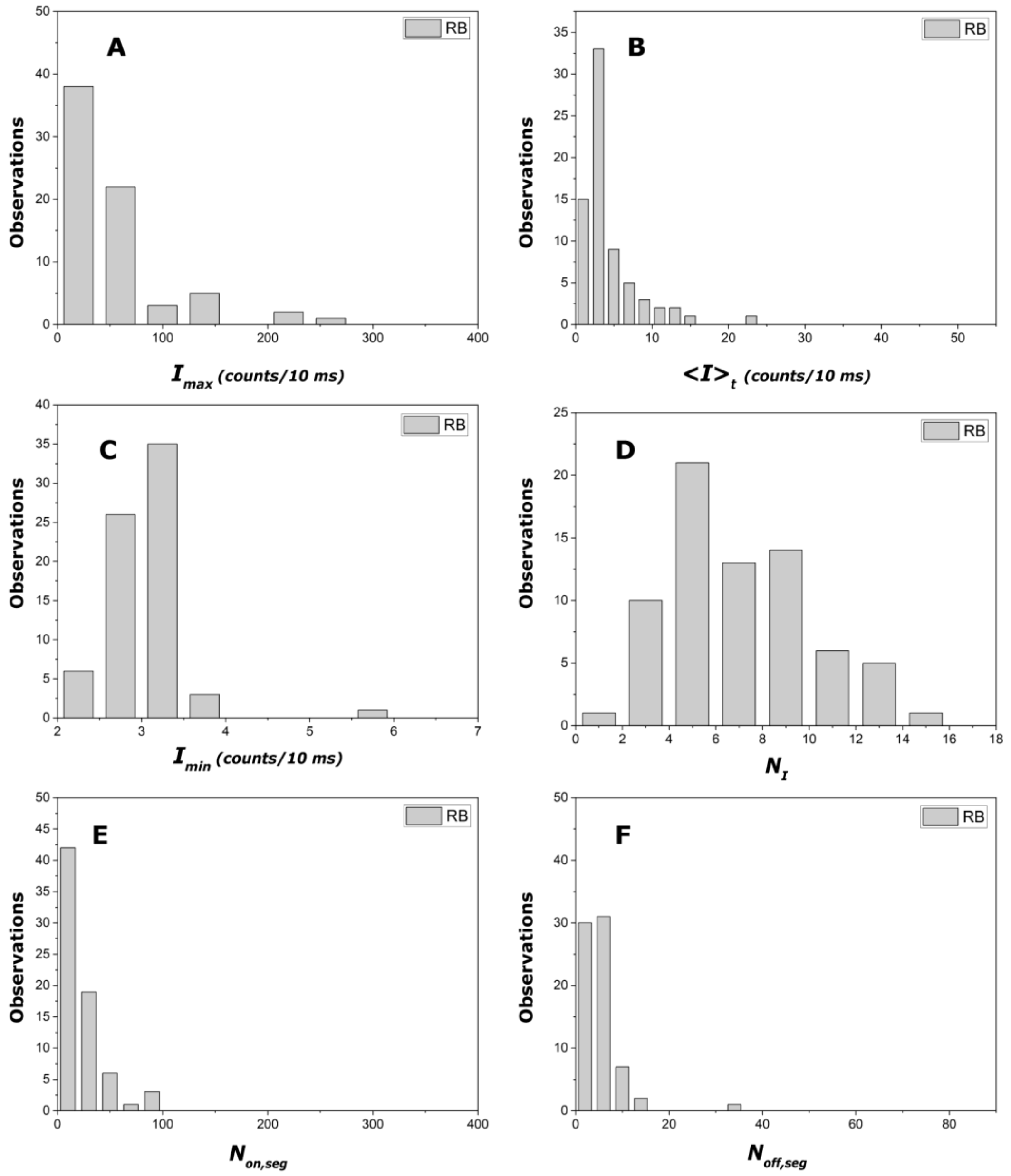

Figure S5. Histograms of (A) I_{max}, (B) $\langle I\rangle_t$, (C) I_{min}, (D) N_I, (E) $N_{on,seg}$, and (F) $N_{off,seg}$ obtained from CPD analysis of 71 RB molecules.

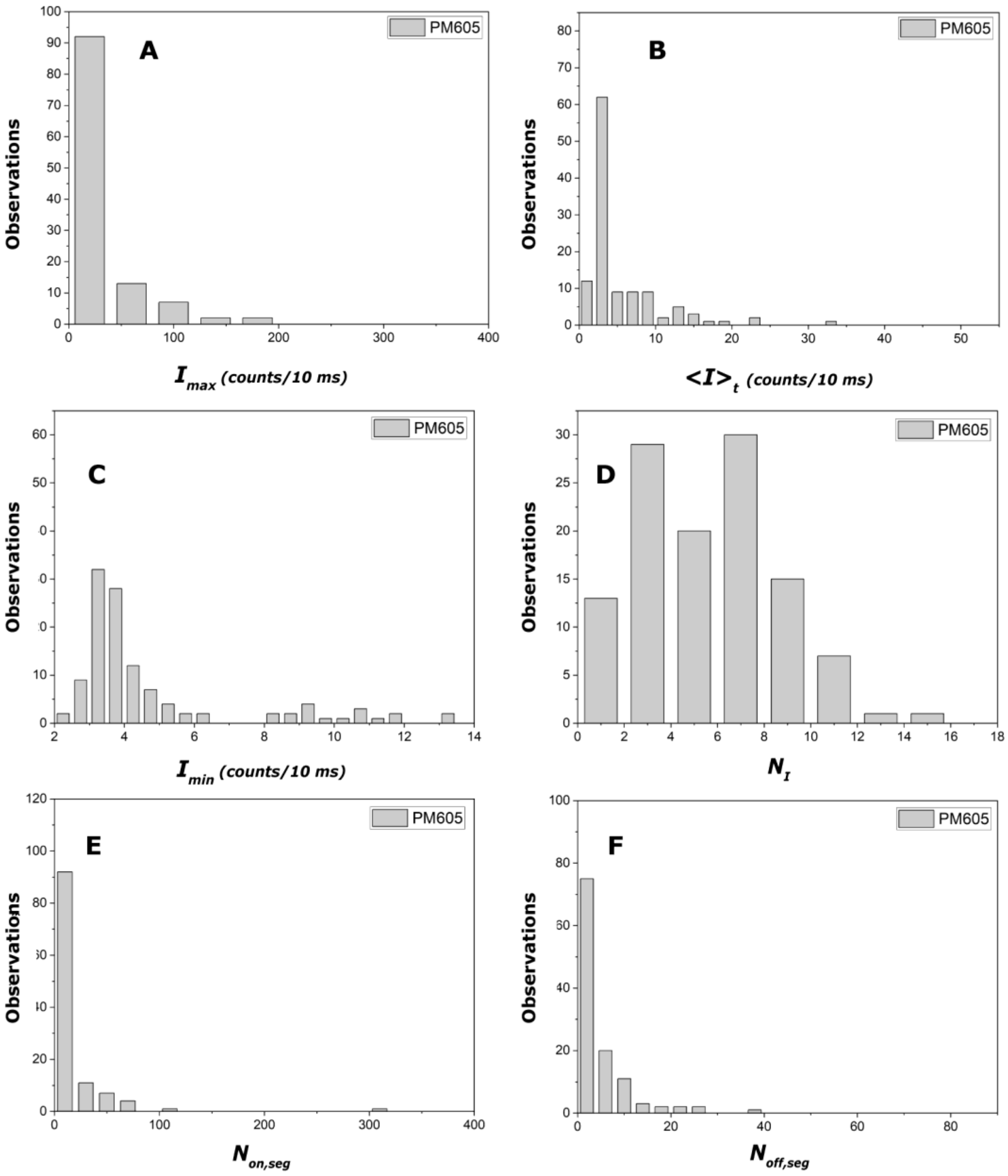

Figure S6. Histograms of (A) I_{max}, (B) $\langle I \rangle_t$, (C) I_{min}, (D) N_I, (E) $N_{on,seg}$, and (F) $N_{off,seg}$ obtained from CPD analysis of 116 PM605 molecules.

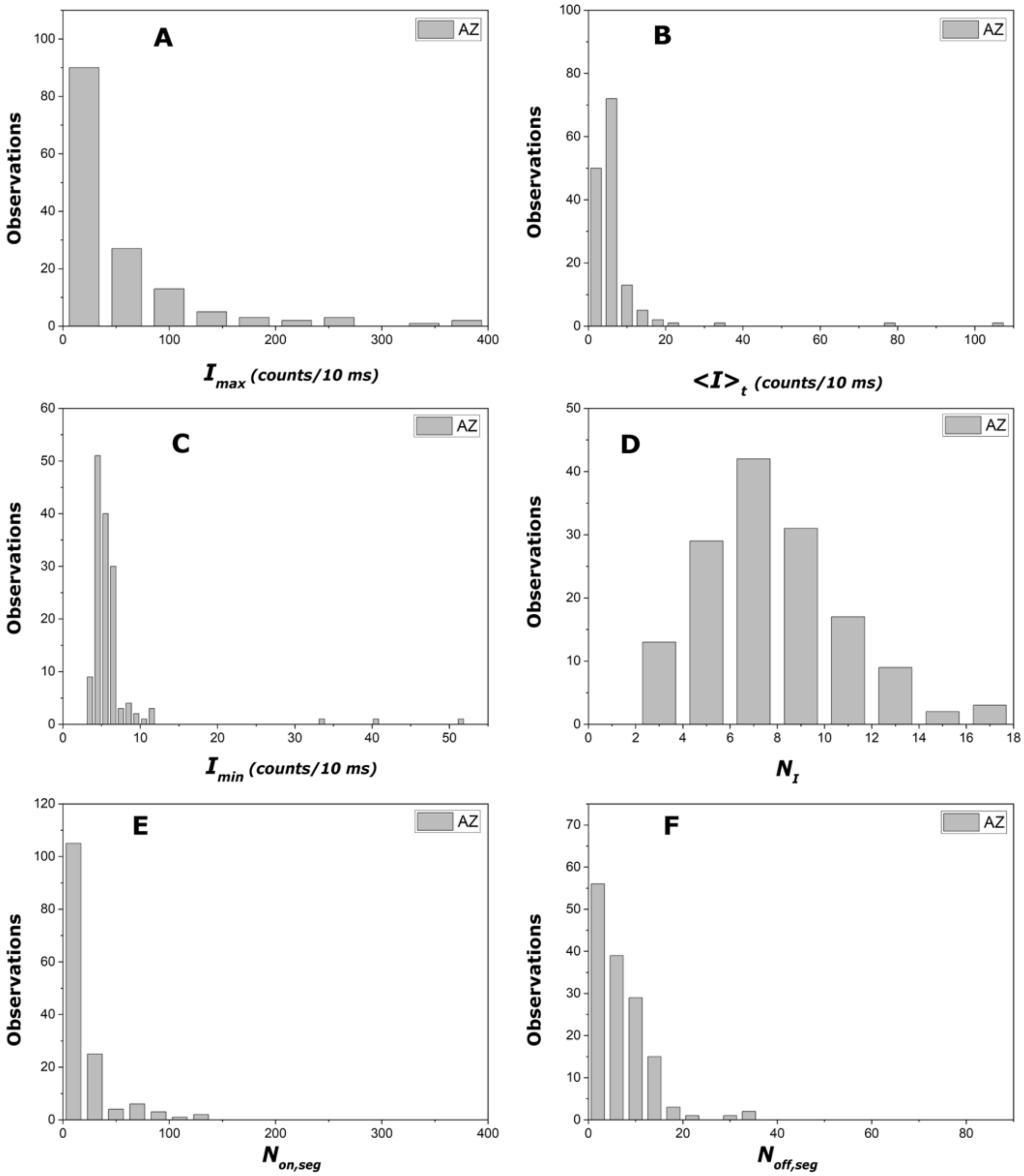

Figure S7. Histograms of (A) I_{max}, (B) $\langle I \rangle_t$, (C) I_{min}, (D) N_I, (E) $N_{on,seg}$, and (F) $N_{off,seg}$ obtained from CPD analysis of 146 AZ molecules. The corresponding distributions of on- and off-event durations and associated fit parameters for AZ are reported in Tan et al. and Table S3, respectively.[1]

Table S2. Best-fit parameters (i.e., regression coefficients and intercept, b) of the 10 CPD-derived blinking statistics and associated identity to sigmoid functions (Eqn. 2) resulting from binary BBM-based classification of 5ROX, R123, R560, R6G, and RB using MLR. Sets in bold represent those that achieve at least 93% accuracy. Although the absolute magnitude of the coefficients cannot be compared across classifications, their relative magnitude within a set report on the statistics governing that particular classification. The largest coefficient within the sets yielding 90% accuracy is highlighted in bold and shows I_{min} is relatively significant. Importantly, we performed control experiments on blank glass substrates to show that variations in I_{min} are not due to systematic differences in optics, excitation power, or experimental conditions – they relate to the minimum emissive intensity, fluorophore brightness, spectral diffusion, as well as the vibrational stability of the experimental setup, which varies randomly across datasets. When I_{min} is excluded as an input predictor for classification, the underlined coefficients have the largest magnitude. The event durations, and in particular $\langle t_{off,int} \rangle$, are important statistics for rhodamine classification, consistent with variations in the average blinking statistics shown in Table S1.

	**5ROX/ R6G**	5ROX/ R123	**5ROX/ R560**	5ROX/ RB	R6G/ R123	R6G/ R560	**R6G/ RB**	R123/ R560	**R123/ RB**	R560/ RB
b	-1.3	-0.3	0.3	0.4	-0.34	-1.1	1.3	0.9	0.8	-0.05
N_I	-0.4	-0.3	-0.9	-1.2	0.1	0.8	-0.5	-0.6	**-0.7**	0.08
$N_{on,seg}$	0.6	0.09	0.4	0.5	-0.03	-0.2	0.4	0.2	0.2	0.3
$N_{off,seg}$	-0.8	-0.3	-0.3	-0.2	-0.3	0.001	0.4	-0.1	0.07	0.06
I_{min}	**-2.6**	-0.5	**-2.6**	-1.2	-1.5	-0.7	**1.5**	-0.5	-0.05	0.8
I_{max}	0.4	0.7	0.3	0.5	0.02	0.5	-0.6	-0.3	-0.3	-0.02
$\langle I \rangle_t$	-0.7	0.2	-0.3	-0.4	-1.2	-1.0	0.6	-0.4	-0.6	-0.4
$t_{on,seg}$	-0.3	0.1	-0.4	0.1	-0.3	-0.4	0.4	-0.3	0.1	0.7
$t_{off,seg}$	-0.2	~0	-0.3	-0.7	-0.2	0.03	-0.4	-0.3	**-0.7**	-0.3
$t_{on,int}$	0.4	0.2	0.4	0.3	0.6	1.1	-0.6	0.03	0.08	-0.3
$t_{off,int}$	0.2	-0.2	-0.2	-0.3	0.4	0.3	-0.7	-0.1	-0.3	-0.3

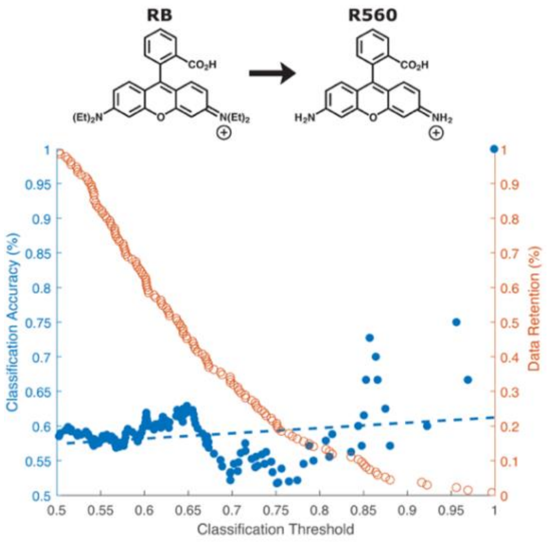

Figure S8. (top) RB undergoes stepwise N-dealkylation at 532 nm to produce R560.[5,6] (bottom) Classification accuracy of RB/R560 is the lowest of all rhodamine trials and plateaus (dashed blue line) at ~60%, even when a classification threshold is applied. Corresponding data retention (red) drops precipitously. BBM does not classify RB/R560, validating the negative control.

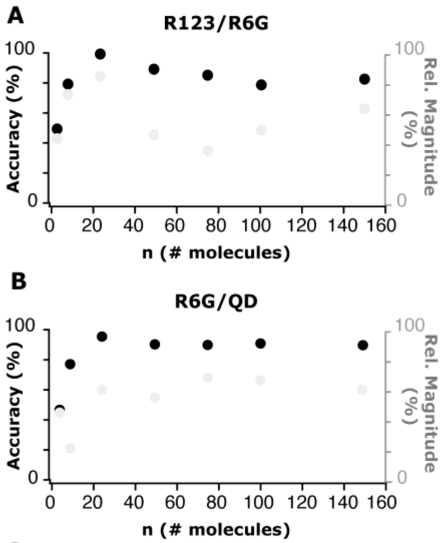

C

% A : % B	
Expected	MLR Predicted
70 QD : 30 R6G	68.0 QD : 32.0 R6G
50 QD : 50 R6G	51.0 QD : 49.0 R6G
50 AZ : 50 R6G	53.3 AZ : 46.7 R6G
30 QD : 70 R6G	41.3 QD : 58.7 R6G

Figure S9. Results of additional MLR analyses and control experiments to validate the classification approach. MLR results of (A) R123/R6G and (B) R6G/QD plotted as a function of number of R6G molecules (*n*) included in training and testing. For example, when 5 R6G and 5 R123 molecules are selected at random for training and testing (*n* = 5), (black circles) minimum classification accuracy is poor (i.e., 50% accuracy, no better than guessing). However, at sufficiently high *n* values of at least 50, both accuracy as well as the identity and (gray circles) total relative magnitude of the 3 highest MLR coefficients stabilize with further increases to *n*. For example, for 75 R123 and 75 R6G molecules (*n* = 75), accuracy is 86 $\pm$ 3%. As *n* is increased to include 100 and then all molecules (i.e., 123 R123 and 148 R6G molecules or *n* = 148), corresponding accuracy values are 80 and 83 $\pm$ 3%, respectively. Error was estimated by varying the molecules selected for training and testing. To further examine the fidelity of BBM via MLR, additional blinking measurements of mixed samples of known composition were performed on several days and by different users. (C) Table of predicted compositions (% class A : % class B) resulting from MLR analysis of 97, 145, 30, and 109 blinking traces of 70:30 [QD]:[R6G], 50:50 [QD]:[R6G], 50:50 [AZ]:[R6G], and 30:70 [QD]:[R6G] mixtures, respectively. Classifications were made without thresholding (i.e., corresponding to default model accuracies of 92.7% and 94.5% for R6G/QD and AZ/R6G, respectively).[4] The predicted compositions for 70:30 [QD]:[R6G], 50:50 [QD]:[R6G], and 50:50 [AZ]:[R6G] are within 1 – 3% of the expected values. The corresponding prediction for 30:70 [QD]:[R6G] is ~11% from the expected value, which is still considered accurate (within sampling, experimental, and classification error).

Table S3. MLE/KS fitting results (i.e., fit parameters and goodness-of-fit *p*-values) for the on- and off-interval durations of the fluorophores included in this study: 5ROX, R123, R560, R6G, RB, PM605, and AZ on glass. Errors represent one standard deviation. The probability that the data matches the hypothetical model is increased as *p* approaches unity. In general, power laws only represent a small subset of the blinking data as evidenced by relatively large t_{min} values, which represent the onset time for power-law behavior. [a]AZ values from Tan et al., which used log-likelihood ratio tests to show that on and off intervals follow lognormal and Weibull distributions, respectively.[1]

		**Power Law:** $\frac{\alpha-1}{t_{min}}\left(\frac{t}{t_{min}}\right)^{-\alpha}$			**Lognormal:** $\frac{1}{t\sigma\sqrt{2\pi}}e^{-\frac{(\ln(t)-\mu)^2}{2\sigma^2}}$			**Weibull:** $\frac{A}{B}\left(\frac{t}{B}\right)^{A-1}e^{-\left(\frac{t}{B}\right)^A}$		
		t_{min} (s)	α	p	μ	σ	p	A	B	p
**ON**	RB	1.65	1.72 ± 0.05	0	0.2 ± 0.1	1.90 ± 0.09	0.65	0.52 ± 0.01	3.0 ± 0.3	0
	R6G	9.75	2.42 ± 0.05	0.31	-0.25 ± 0.07	1.95 ± 0.05	0.007	0.58 ± 0.01	2.0 ± 0.1	0
	5ROX	7.31	2.2 ± 0.1	0.39	0.0 ± 0.1	1.77 ± 0.09	0.48	0.58 ± 0.01	2.5 ± 0.3	0.03
	R123	9.59	2.6 ± 0.1	0.81	0.00 ± 0.01	1.94 ± 0.08	0.13	0.53 ± 0.01	2.4 ± 0.2	0
	R560	5.57	1.99 ± 0.06	0.003	0.1 ± 0.1	2.02 ± 0.08	0.099	0.51 ± 0.01	3.0 ± 0.3	0
	PM605	0.79	1.57 ± 0.03	0	0.3 ± 0.1	2.01 ± 0.08	0.81	0.50 ± 0.01	3.5 ± 0.3	0
	AZ[a]	9.2	2.44 ± 0.07	0.04	0.81 ± 0.08	1.58 ± 0.06	0.07	0.68 ± 0.01	5.0 ± 0.4	0
**OFF**	RB	0.03	1.29 ± 0.03	0	-0.3 ± 0.2	2.6 ± 0.1	0.16	0.38 ± 0.01	2.9 ± 0.3	0
	R6G	3.35	1.88 ± 0.03	0.21	-1.27 ± 0.08	2.07 ± 0.05	0	0.47 ± 0.01	0.87 ± 0.05	0
	5ROX	0.52	1.68 ± 0.05	0.15	-1.3 ± 0.2	2.0 ± 0.1	0.03	0.51 ± 0.02	0.9 ± 0.1	0
	R123	2.76	1.65 ± 0.04	0.002	-0.1 ± 0.1	2.3 ± 0.1	0.19	0.44 ± 0.01	2.8 ± 0.3	0
	R560	0.76	1.52 ± 0.03	0.006	-0.7 ± 0.1	2.4 ± 0.1	0.012	0.43 ± 0.01	1.8 ± 0.2	0
	PM605	10.7	2.43 ± 0.08	0.77	-0.7 ± 0.1	2.04 ± 0.08	0.03	0.50 ± 0.01	1.4 ± 0.1	0
	AZ[a]	65.3	10.5 ± 0.5	0.09	1.65 ± 0.09	1.74 ± 0.06	0	0.62 ± 0.1	12.0 ± 0.9	0

Table S4. Best-fit parameters resulting from binary BBM-based classification of 1 rhodamine (Rh) (i.e., 5ROX, R123, R560, R6G, or RB) versus PM605 or AZ using MLR. The relative magnitude of the coefficients within a set report on the statistics governing classification. The largest coefficient is presented in bold, which shows I_{min} is relatively significant for classification against PM605 or AZ, except for PM605/R6G. The next most important statistics for classification are underlined. In addition to I_{min}, the off-segment durations are consistently important for PM605/Rh classification, consistent with differences in the average $\langle t_{off,seg} \rangle$ as shown in Table S1. For classification against AZ, $\langle I_t \rangle$ consistently is second most important, consistent with ESIPT acting to protect AZ from photobleaching and thereby increasing its time-averaged intensity.[1] The durations of on and off events are also relatively important.

	**5ROX/ PM605**	**PM605/ R6G**	**PM605/ R123**	**PM605/ R560**	**PM605/ RB**	**AZ/5ROX**	**AZ/R6G**	**AZ/R123**	**AZ/R560**	**AZ/RB**	**AZ/ PM605**
b	-5.6	-0.4	0.6	1.5	2.2	-5.1	1.2	2.0	2.5	201.4	0.4
N_I	-1.5	0.1	1.3	-0.8	-0.1	-1.0	1.6	0.9	0.9	9.3	**1.1**
$N_{on,seg}$	1.8	-0.2	-1.3	-0.2	-0.6	0.2	-1.6	-0.6	-0.5	-15.1	-0.75
$N_{off,seg}$	0.3	-0.8	-0.7	-0.7	-0.5	0.7	-0.1	0.2	0.2	44.1	0.78
I_{min}	**-11.2**	0.7	**4.0**	**1.8**	**4.6**	**-16.0**	**13.7**	**15.9**	**9.8**	**815.5**	**1.1**
I_{max}	1.1	-0.1	-0.6	-0.9	-1.0	1.0	0.8	-0.7	0.0	18.7	0.4
$\langle I \rangle_t$	-0.7	-0.1	1.3	1.0	0.6	1.6	-5.6	-1.3	-3.4	-217.7	**1.1**
$t_{on,seg}$	-0.4	0.0	0.1	0.2	0.0	0.0	0.1	0.4	0.6	6.4	-0.04
$t_{off,seg}$	2.2	**-1.4**	-1.3	-1.5	-2.1	-0.5	0.5	1.1	0.7	55.5	0.6
$t_{on,int}$	0.8	0.1	-0.9	-1.1	-0.7	-0.4	0.7	-0.5	-1.0	-3.7	0.09
$t_{off,int}$	-0.2	0.2	-0.4	-0.2	-0.6	-1.5	-0.2	-0.7	-0.2	-25.2	0.2

Table S5. MLE/KS fitting results for the on- and off-segment durations of 5ROX, R123, R560, R6G, RB, and PM605 on glass to lognormal functions. Errors represent one standard deviation.

		**Lognormal:** $\frac{1}{t\sigma\sqrt{2\pi}} e^{-\frac{(\ln(t)-\mu)^2}{2\sigma^2}}$		
		μ	σ	p
**ON**	RB	-1.39 ± 0.05	1.71 ± 0.03	0
	R6G	-1.79 ± 0.03	1.65 ± 0.02	0
	5ROX	-1.66 ± 0.05	1.58 ± 0.04	0
	R123	-1.30 ± 0.05	1.64 ± 0.03	0.003
	R560	-1.65 ± 0.04	1.67 ± 0.03	0
	PM605	-1.11 ± 0.04	1.80 ± 0.03	0
**OFF**	RB	-1.39 ± 0.05	2.8 ± 0.1	0
	R6G	-0.09 ± 0.08	2.68 ± 0.06	0
	5ROX	0.3 ± 0.2	3.0 ± 0.1	0
	R123	1.2 ± 0.1	2.43 ± 0.07	0
	R560	0.4 ± 0.1	2.66 ± 0.09	0
	PM605	0.2 ± 0.1	2.35 ± 0.07	0.023

Table S6. Ternary BBM-based classification of 5 Rh (i.e., 5ROX, R123, R560, R6G, RB), PM605, AZ, and QD emitters arranged by (1) classification type and (2) corresponding minimum accuracy. Minimum accuracy corresponds to a default threshold (i.e., $P_A > 0.33$ is classified as A). Subsequent columns contain the classification threshold and corresponding data retention values (i.e., for emitters in class A, B, and C, as well as overall). Sets in bold yield the best BBM performance (i.e., high minimum accuracy and 90% model accuracy with overall data retention >50%). Blank cells indicate the desired accuracy is not achieved with at least 10% data retention.

Type	Specific Classification (A/B/C)	Minimum accuracy (%)	for 90% accuracy					for 93% accuracy				
			threshold	retention, A (%)	retention, B (%)	retention, C (%)	overall retention (%)	threshold	retention, A (%)	retention, B (%)	retention, C (%)	overall retention (%)
AZ/Rh/QD	**5ROX/AZ/QD**	89.2	0.5	99.3	100.0	98.6	99.3	0.6	94.8	87.7	88.8	90.3
	**AZ/QD/RB**	88.1	0.6	97.3	95.8	93.0	95.8	0.7	87.7	88.8	83.1	87.2
	**AZ/QD/R123**	86.5	0.6	88.4	90.9	95.5	91.5	0.7	73.3	81.8	88.0	80.8
	**AZ/QD/R560**	85.8	0.7	79.5	88.1	71.9	81.6	0.8	53.4	78.3	39.1	60.9
	**AZ/QD/R6G**	77.8	0.7	59.6	81.1	62.8	67.7	0.9	38.4	71.3	40.5	49.9
2Rh/QD	**5ROX/QD/R560**	85.1	0.7	69.5	93.0	51.6	76.8	0.9	41.1	89.5	32.8	62.3
	**5ROX/QD/R6G**	84.7	0.7	76.8	90.9	90.5	87.3	0.9	16.8	77.6	51.4	52.6
	QD/R123/R6G	84.4	0.9	71.3	7.6	14.9	31.7	0.9	69.9	5.3	9.5	28.6
	**QD/R6G/RB**	83.4	0.7	90.9	77.7	32.4	74.0	0.9	77.6	35.8	15.5	48.3
	**5ROX/QD/RB**	80.9	0.7	65.3	99.3	43.7	76.1	0.8	44.2	99.3	21.1	64.1
	**QD/R123/RB**	79.5	0.7	93.0	70.5	36.6	72.8	0.8	90.9	46.2	22.5	59.8
	**QD/R123/R560**	79.4	0.8	90.2	59.1	34.4	67.6	0.8	89.5	55.3	23.4	63.7
	**QD/R560/R6G**	75.2	0.8	81.1	15.6	41.2	52.7	0.9	76.9	6.3	27.0	43.4
	**5ROX/QD/R123**	73.5	0.7	13.7	95.8	20.5	47.8	0.8	8.4	93.7	10.6	42.2
2Rh/AZ	**5ROX/AZ/R560**	80.6	0.8	69.6	79.5	32.8	67.0	0.9	33.3	65.1	15.6	43.5
	**5ROX/AZ/RB**	78.4	0.8	44.4	89.7	23.9	59.1	0.8	24.4	88.4	9.9	48.0
	**5ROX/AZ/R6G**	77.9	0.7	72.6	71.2	47.3	63.4	0.9	7.4	48.6	9.5	22.1

	Specific Classification (A/B/C)	Minimum accuracy (%)	for 90% accuracy					for 93% accuracy				
			threshold	retention, A (%)	retention, B (%)	retention, C (%)	overall retention (%)	threshold	retention, A (%)	retention, B (%)	retention, C (%)	overall retention (%)
	**AZ/R123/RB**	77.7	0.8	86.3	57.9	31.0	64.3	0.9	84.2	12.0	12.7	42.3
	AZ/R123/R6G	76.8	0.9	51.4	10.5	10.8	24.6	0.9	50.0	6.8	8.8	22.2
	**AZ/R123/R560**	76.4	0.7	80.1	79.7	39.1	72.3	0.8	71.2	66.9	21.9	60.3
	AZ/R6G/RB	73.4	0.8	67.8	23.0	16.9	39.7	0.9	55.5	10.8	2.8	27.1
	5ROX/AZ/R123	71.0	0.7	17.8	82.9	6.8	37.2	0.8	11.9	78.1	4.5	32.9
	AZ/R560/R6G	67.3	0.8	59.6	15.6	15.5	33.5	0.8	53.4	9.4	9.5	27.4
2Rh/PM605	5ROX/PM605/R560	72.7	0.9	20.0	34.5	7.8	23.3					
	PM605/R123/RB	71.2	0.9	31.0	0.8	4.2	12.5					
	**5ROX/PM605/RB**	70.9	0.8	41.1	68.1	22.5	47.5	0.9	14.7	46.6	4.2	25.2
	PM605/R123/R6G	70.2										
	5ROX/PM605/R6G	69.9										
	5ROX/PM605/R123	64.4										
	PM605/R123/R560	67.3	0.8	32.8	18.9	7.8	21.8					
	PM605/R6G/RB	65.4										
	PM605/R6G/R560	61.3										
3Rh	5ROX/R123/R6G	64.3										
	R123/R6G/RB	68.9										
	5ROX/R6G/RB	70.1	0.9	3.2	29.7	1.4	15.3					
	5ROX/R560/R6G	69.7										
	R123/R560/R6G	66.3										
	5ROX/R123/R560	53.3										
	5ROX/R123/RB	52.7										

Supplementary References

1. Tan, J. *et al.* Contributions from Excited-State Proton and Electron Transfer to the Blinking and Photobleaching Dynamics of Alizarin and Purpurin. *J. Phys. Chem. C* **121**, 97–106 (2017).
2. Amat, A., Miliani, C., Romani, A. & Fantacci, S. DFT/TDDFT investigation on the UV-vis absorption and fluorescence properties of alizarin dye. *Physical Chemistry Chemical Physics* **17**, 6374–6382 (2015).
3. Lee, S., Lee, J. & Pang, Y. Excited state intramolecular proton transfer of 1,2-dihydroxyanthraquinone by femtosecond transient absorption spectroscopy. *Curr. Appl. Phys.* **15**, (2015).
4. Hoy, G. R., DeSalvo, G. A., Haile, S. H., Smith, E. N. & Wustholz, K. L. Rapid, Accurate Classification of Single Emitters in Various Conditions and Environments for Blinking-Based Multiplexing. *J. Phys. Chem. A* **127**, 3518–3525 (2023).
5. Cassidy, J. P., Tan, J. A. & Wustholz, K. L. Probing the Aggregation and Photodegradation of Rhodamine Dyes on TiO2. *J. Phys. Chem. C* **121**, 15610–15618 (2017).
6. Chen, F., Zhao, J. & Hidaka, H. Highly selective deethylation of Rhodamine B: Adsorption and photooxidation pathways of the dye on the TiO2/SiO2 composite photocatalyst. *Int. J. Photoeng.* **5**, 209–217 (2003).
